# Supplementary material for: Utilizing cost-effective pyrocarbon for highly efficient gold retrieval from e-waste leachate
Source: Nat Commun. 2024 Jul 20;15:6137. doi: 10.1038/s41467-024-50595-4 (PMC11271467; doi:10.1038/s41467-024-50595-4)
Supplement: Supplementary file 1 — Supplementary Information [file 41467_2024_50595_MOESM1_ESM.pdf]

Supplementary Information for

Utilizing Cost-Effective Pyrocarbon for Highly Efficient Gold Retrieval from E-Waste Leachate

Fu et al.

This PDF file includes:

Supplementary Method. 1 to 7

Supplementary Fig. 1 to 52

Supplementary Table. 1 to 11

Supplementary Methods

Supplementary Method 1. Materials and chemicals.

Sodium alginate (SA, ≥ 99.0%), calcium chloride ($CaCl_2$, ≥ 97.0%), aluminum chloride ($AlCl_3{\cdot}6H_2O$, ≥ 97.0%), copper nitrate ($Cu(NO_3)_2{\cdot}3H_2O$, ≥ 99.0%), hydrochloric acid (HCl, 37.0%), sodium hydroxide (NaOH, ≥ 98.0%), nickel nitrate ($Ni(NO_3)_2{\cdot}6H_2O$, ≥ 98.0%), zinc nitrate ($Zn(NO_3)_2{\cdot}6H_2O$, ≥ 99.0%), potassium tetrabromoaurate ($KAuBr_4$, ≥ 99.9%)), sodium bicarbonate ($NaHCO_3$, ≥ 99.5%), sodium carbonate (Na_2CO_3, ≥ 99.0 %), ferric chloride ($FeCl_3{\cdot}6H_2O$, ≥ 99.0%), sodium fluoride (NaF, ≥ 98.0%), sodium chloride (NaCl, ≥ 99.0%), sodium bromide (NaCl, ≥ 99.0%), potassium iodide (NaI, ≥ 99.0%), sodium sulfate (Na_2SO_4, ≥ 99.0%), and potassium phosphate (K_3PO_4, ≥ 98.0%) were purchased from Sinopharm Chemical Reagent Co., Ltd., Shanghai, China. Gold(III) chloride ($HAuCl_4$, ≥ 99.0%), Gold(I) potassium cyanide ($KAu(CN)_2$, ≥ 99.5%), N-Bromo succinimide (NBS, ≥ 99%) and Pyridine (Py, ≥ 99%) were purchased from Aladdin Chemical Co., Ltd. The discarded Advanced Micro Devices (AMD) and Intel central processing units (CPUs) were purchased from a local computer repair shop in Shanghai, China. A Milli-Q ultrapure water purification system (Millipore, Billerica, MA, resistivity > 18.2 MΩ·cm) was used to produce deionized water (DIW).

Supplementary Method 2. Analysis and characterizations.

Prior to the characterizations, the pyrocarbon samples were activated in a vacuum drier for 24 h. The morphologies and elemental distributions of the samples were obtained using scanning electron microscope (SEM, MIRA3 TESCAN). The crystallographic information was collected on an X-ray diffractometer with Cu-Kα radiation source (XRD, Smartlab). Raman spectra were measured using a Raman spectrometer with a 532 nm laser as the excitation source (Horiba Scientific LabRAM HR Evolution). The N_2 adsorption isotherms were measured at 77 K using a surface

area analyzer (JW-BK200C, JWGB SCI.&TECH). Fourier transform infrared spectra (FTIR) measurements were performed from KBr using IR spectrometer (Thermo Scientific Nicolet iS5, Thermo Fisher Scientific). The optical photograph of the recycled gold particles was obtained with a laboratory light microscope. The surface charge of the pyrocarbon was analyzed using system zeta potential analyzer (Litesizer 500, Anton Paar). Concentration of persistent free radicals within pyrocarbon samples were analyzed by electron paramagnetic resonance (EPR) spectroscopy (Micro ESR, Bruker).

Supplementary Method 3. Adsorption modeling and thermodynamic calculations.

(1) To calculate the maximum recovery capacity of Au(III) on pyrocarbon, the equilibrium isotherm data are fitted by Langmuir model using non-linear equation:

$$q_e = \frac{q_m k_L C_e}{1 + k_L C_e} \quad (1)$$

where q_m (mg g^{-1}) is theoretical maximum recovery capacity, k_L (L mg^{-1}) is the corresponding adsorption equilibrium constants.

(2) Thermodynamics were calculated using the equations:

$$\Delta G^{\circ} = -RT \ln K \quad (2)$$

$$\ln K = \frac{\Delta S^{\circ}}{R} - \frac{\Delta H^{\circ}}{RT} \quad (3)$$

where R is the universal gas constant (8.314×10^{-3} kJ $(mol \cdot K)^{-1}$) and T is the temperature (K), K is the dimensionless equilibrium constant derived from Langmuir equilibrium constant k_L.[1]

(3) The kinetics were described using mass transfer model. In general, the mass flow rate of Au(III) is calculated using the following equation:

$$-\frac{dC}{dt} = k_f a (C - C_s) \quad (4)$$

where k_f is the mass transfer coefficient (m s^{-1}), a is the effective area for mass transfer per unit volume of the contactor, C is the concentration of Au(III) in solution, and C_s is the concentration of Au(III) at adsorbent surface.

In the Langmuir model, the adsorption capacity (q_e) can be calculated using the equation:

$$q_e = \frac{V(C_0 - C_s)}{m} \tag{5}$$

By combining equation 1, 4 and 5, equation 6 can be obtained:

$$-\frac{dC}{dt} = k_f \, a \, [C - \frac{1}{2}(\sqrt{(\frac{q_m m}{V} - C_0 + \frac{1}{k_L})^2 + \frac{4C_0}{k_L}} - \frac{q_m m}{V} + C_0 - \frac{1}{k_L})] \tag{6}$$

According to equation 6, we can derive the Au(III) concentration (C) as a function of time (t) to describe the adsorption kinetics:

$$C = b\exp[-ht] + C_0 - b \tag{7}$$

where h is the fitting parameter, representing $k_f a$. Therefore, equation 7 can be applied to fit experimental data, and to estimate k_f. In this model, calculating k_f values incorporated a critical normalization procedure that considered various reaction details (e.g., specific surface area, sorbent dosages) during sorption processes. It was widely employed in previous studies because it can effectively and adequately explain mass transfer phenomena and offers valuable insights into adsorbate diffusion and transfer. Furthermore, it accurately represents the mass transfer occurring within porous structures.[2-4]

Supplementary Method 4. Preparation of gold(III) containing CPU leachate.

For the aqua regia leaching method: The CPU scraps were soaked in 50 mL NaOH solution (8 mol L^{-1}) for 2 days to dissolve the protective resin on the scrap surfaces. The treated scraps were then rinsed with tap water and submerged in 50 mL aqua regia at 60°C for 2 days. Regarding the NBS-Py leaching method: The NBS-Py leaching solution was prepared by dissolving 0.8 mL pyridine and 0.89 g NBS into 100 mL DIW. Subsequently, the CPU scraps were soaked in the leaching solution for 24 h. After leaching processes, the undissolved solids were filtered out and rinsed with DIW to form the resultant CPU leachate. Considering that the aqua regia has strong oxidation properties that can reduce the reducing ability and adsorbent performance significantly, the AMD-Aqua leachate was also subjected to a 10-fold

dilution in order to mitigate the acidity issues within the solution, which was labeled as AMD-Aqua*10. Alkali neutralization was not employed due to its impracticality when scaled up since instant precipitate formation occurs.

Supplementary Method 5. Electrochemical analysis tests.

(1) Open circuit potential (OCPT) measurement.

First, a certain amount of PyC700 granulates were fully grinded with an agate mortar. Then 4 mg PyC700 was added into 480 μL isopropanol solution, followed by ultrasonic treatment for 1 h to increase its dispersity in isopropanol. Afterward, 50 μL 5% Nafion perfluorinated resin was added into the PyC700 solution. After stirring overnight, the uniform PyC700 ink was dipped on a polished glassy carbon electrode (GCE) and placed into an oven at 60°C for 10 min. A PyC700-coated glassy carbon electrode (PyC700-GCE) was prepared after repeating the dipping step three times (4.5 μL ink in total). The PyC700-GCE was dipped in the DIW with pH 1.0 overnight to maintain a stable potential before electrochemical analysis. Then the open circuit potential of PyC700-GCE was monitored by OCPT analysis in a three-electrode-cell configuration including an Ag/AgCl electrode as the reference electrode and a platinum mesh as the counter electrode. After the OCPT of PyC700-GCE being stabilized, 1 mL of different metal ions solution (5 mM) was added into the electrochemical cell containing 60 mL of 0.5 M Na_2SO_4 to monitor the potential variation of the system.

(2) Electron accepting capacities (EACs) and donating capacities (EDCs) test.

The EACs and EDCs properties of pyrocarbon samples were quantified using the mediated electrochemical reduction (MER) and oxidation (MEO) approach, respectively.[5] Specifically, a 9 mL glassy carbon cylinder served both as the working electrode (WE) and electrochemical reactor, and the platinum electrode, and Ag/AgCl electrode were used as counter electrode and reference electrode, respectively, in the electrochemical workstation. In this approach, an electrochemical cell (glassy carbon cylinder) containing a pH-buffered solution is set to a constant E_H^- value while the

current is measured over time. In the presence of a mediator in redox equilibrium with WE, known amounts of a sample are added to the electrochemical cell, resulting in current responses that can be integrated to directly determine the number of electrons transferred to or from WE. The MER and MEO were conducted at an applied potential of -0.49 and +0.61 V, respectively. 100 μL solutions (10 mmol L^{-1}) of electron transfer mediators ZiV (in MER) or ABTS (in MEO) were added to the electrochemical cell filled with 5 mL of phosphate buffer (0.1 mol L^{-1} phosphate and 0.1 mol L^{-1} KCl, pH 7.0). When the current became constant, 100 μL of pyrocarbon suspensions (2.0 g L^{-1}) were spiked into the cell, resulting in the reductive or oxidative current peaks (This step was conducted 4 times to test the four pyrocarbon samples). Integration of current peaks yielded the EAC and EDC [mmol e^{-} g^{-1}] of pyrocarbon.

Supplementary Method 6. Trend analysis between gold adsorption performance and pyrocarbon properties.

According to the available characterization results, an increase in the preparation pyrolytic temperature led to enhanced specific surface area (SSA), conductivity, structural defects, and graphitization degree of the PyCs. Both higher SSA and conductivity are advantageous for gold recovery performance of PyCs due to their ability to facilitate mass transfer and electron transfer on carbon matrices, thereby promoting the reduction of targeted Au(III) ions into Au^0. However, adsorption results indicate volcano-shaped diagrams for Q_m and k_f, with PyC700 exhibiting optimal adsorption capacity and kinetics. This suggests that other critical properties in pyrocarbon significantly influence gold recovery performance. The XPS and FTIR results revealed a notable decline in surface functionalities of PyCs at higher pyrolysis temperatures, as evidenced by the decreasing oxygen contents from PyC500 to PyC800. The Boehm titration and electrochemical tests revealed that the amounts of phenolic -OH and the associated electron-exchange capacity were found to decrease with the pyrolysis temperatures increase. Therefore, it can be speculated that a

trade-off exists within various characteristics of PyCs (e.g., surface functionality, porosity, conductivity, and electron-exchange capacity) that results in the gold recovery performance increasing first and then decreasing with the increase in pyrolysis temperatures of the prepared PyCs. The trend was similarly observed by Cheng and colleagues,[6] who reported the highest extraction capacity of Au(III) for reduced graphene oxide (with a moderate oxidation degree). In contrast, graphene oxide (with the highest oxidation degree), commercial graphene, and expanded graphite (with the lowest oxidation degree) exhibited significantly lower extraction capacities. Although their studies did not provide a molecular understanding to the electrons donation mechanism, their results validate the trade-off we reported here. Both sp^2-hybridized carbon in graphite-like structure and sp^3-hybridized carbon in the amorphous region associated with oxygen-containing groups are crucial for gold recovery capacity of pyrocarbon.

Supplementary Method 7. Details of technoeconomic analysis (TEA).

To evaluate the economic potential for Au(III) recovery based on pyrocarbon adsorption, a TEA of the whole gold recovery process was carried out to demonstrate a diagram for applying pyrocarbon adsorbent in actual manufacturing industry. Primarily, material flow of the entire gold recovery processes based on PyC700 was analyzed, where three main steps such as CPU leaching, adsorbent production, and gold separation/purification were included. The general steps of CPU leaching and gold separation/ purification can be easily scaled up because of their simplicity and factual cases. By referring to the Guidelines for Techno-Economic Analysis of Adsorption Processes written by Danaci et al,[7] the production step of PyC700 could meet the industrial suitability, taking into account the required characteristics (e.g., water tolerance, rapid mass transfer, low cost, low environmental and safety impact, etc.). The material ratios, reaction parameters (working temperature, reaction time, etc.), and the yield of the products employed during TEA were determined with reference to laboratory scale realities. We have summarized the technological

parameters of the electric equipment during the gold recovery processes in Supplementary Fig. 51. The methods for the leaching of CPU scrap and the purification of recovered gold were referenced from previous literature.[6] Of note, the electricity price selected in this work (0.02 ~ 0.06 \$ kWh^{-1}) was based on the recently published literature in which TEA were carried out for electrosynthesis of ethylene.[8] Notably, the electricity cost in our work were found to be much lower than the material and labor cost, and showed negligible sensitivity in sensitivity analysis, which corresponded to the previous studies.[9] Labor costs are estimated on the basis of the average monthly salary of workers globally in 2022 (including some low- and high-income countries). It was found that monthly workers' salaries showed a secondary sensitivity among the four variables, and that the impact of monthly workers' salaries on total costs may be greater in the case of hiring more workers. It can be surmised that the rational location of production plants is important for cost control and profitability. However, the cost of CPU scrap is an important part of the total cost of gold recovery process, whereas the CPU scrap varies in price and gold content depending on the type of scrap, in addition to the choice of recycling channel (retailers, hawkers, recycling organizations), which can cause the price to fluctuate. On the basis of both the CPU scrap on the market and also the literature reported values,[10, 11] the gold content in the CPU scrap is considered to be 5 grams per kilogram, and its price has been selected at US$ 13.72 per kilogram, which is higher than the wholesale purchase price.

The specific sample for the calculation of total cost of the proposed gold recovery process from CPU scrap using pyrolytic PyC700.

To specify the economic potential of the gold recovery process using PyC700 we proposed, a 7.3 kilograms annual production of PyC700 with 20.7 kilograms of gold being recovered was took as an example. In accordance with the actual experimental conditions during our lab-scale production of PyC700, the calculation details such as material ratios and reaction parameters were carefully selected. Specifically, we assumed a daily output of 20 g day^{-1} for the sorbents, resulting in an annual

production of 7.3 kilograms for PyC700. The annual running times for the equipment were determined based on their actual working time in preparation processes, which are provided in Supplementary Fig. 51. The specific assumptions have been discussed in the following texts for cost calculations. The material flow with subdivided costs for the entire gold recovery process was analyzed, which could provide some reference and guidance, and of course, the actual profit must be determined after a large-scale.

(1) Capital cost. The total capital cost was considered to include the equipment purchase cost and other capital costs. To be noted, the other capital costs were typically generated due to equipment installation, process piping, instrumentation and controls, electrical systems, etc. These costs are difficult to determine as they depend on many factors, and some approximations exist to estimate these costs as a percentage. To ensure the accuracy of the results, we have referred to the methods previously reported to calculate the capital costs for producing honeydew peel activated carbon.[12]

1. Equipment purchase cost:

   = \$27.43 (laboratory water bath)+\$274.35 (peristaltic pump)+\$20.58 (Magnetic stirrer)+\$685.87 (freeze dryer)+\$1646.09 (tube furnace)+\$548.70 (thermostatic oscillator)+\$41.2 (glass wares)=\$3244.17

2. Other capital costs: The other capital costs were calculated by assuming them 57.84% of the total fixed capital estimate as reported by Yunus and coworkers.[12] Therefore, the other capital costs can be calculated to be \$4450.73.

(2) Operating costs. The operating costs were considered to include the electricity, maintenance, labor, depreciation, and other operating costs (e.g., administrative cost, insurance fee, etc.). The selected electricity price (0.06 \$ kWh^{-1}) was based on local electricity price in Shanghai, China, a combination of peak- and valley-time prices. Annual maintenance materials were considered as 2.0% of the total equipment cost.[13] The labor cost was calculated based on the average wage in China in 2022. Depreciation is an accounting manner, the return of fixed capital estimate. As

suggested in technoeconomic analysis for rice husk adsorbent production,[14] we have calculated the depreciation by assuming the 10% annual of the fixed capital. The administrative cost including expenses incurred for the support of production (administrative staff, offices, sales network, etc.) were considered as 45.0% of the labor cost.[14] Insurance fee referring to the plant site and the product was calculated as 1.0% of the fixed capital.[14]

1. Electricity:

   = (0.3 kW×2 h day^{-1}+0.02 kW×6 h day^{-1}+0.2 kW×6 h day^{-1}+1.6 kW×24 h day^{-1}+1.2 kW×6 h day^{-1}+0.38 kW×12 h day^{-1})×365 day×0.06 \$ kWh^{-1}=\$1140.55

2. Maintenance:

   = \$3244.17 (equipment purchase cost)×2.0%=\$64.88

3. Labor:

   =1113.08 \$ $month^{-1}$×12 month=\$13356.96

4. Depreciation:

   = \$7694.90×10.0%=\$769.49

5. Other operating cost:

   =1113.08 \$ $month^{-1}$×12 month×45.0% (administrative cost)+ \$7694.90×1.0% (Insurance fee)=\$6087.58

(3) Material costs. The prices of all the raw chemicals and reagents were based on industrial grade purity reagents, and were obtained from the recent prices on the official website of Baidu Alibaba International Shopping (https://b2b.baidu.com), the local suppliers, and also the previous studies.[9] The annual usage of these chemicals were calculated based on their daily consumption in the lab-scale production processes, which are provided in Supplementary Fig. 51.

1. NaOH:

   =1194.70 \$ t^{-1}×0.291 t=\$348.25

2. Sodium alginate:

   =10973.93 \$ t^{-1}×0.071 t=\$775.51

3. CPU scrap:

=13717.42 \$ t^{-1}×4.132 t=\$56677.64

4. Other chemicals ($CaCl_2$, HCl, HNO_3, Water):

   = (68.58 \$ t^{-1}×0.292 t)+(40.47 \$ t^{-1}×0.693 t)+(205.76 \$ t^{-1}×0.755 t)+(0.8 \$ t^{-1}×29.2 t)=\$226.83

(4) Total cost.

Total cost=(\$3244.17+\$4450.73)+(\$1140.55+\$64.88+\$13356.96+\$769.49+\$6087.58) +(\$348.25+\$775.51+\$226.83+\$56677.64)= \$87142.59

Supplementary Figures

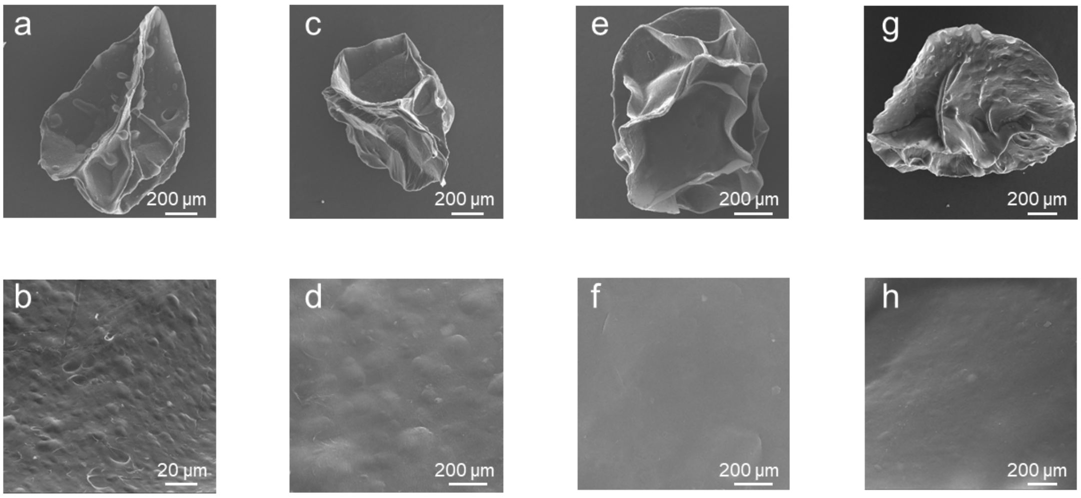

Supplementary Fig. 1. SEM images of the synthesized (a, b) PyC500, (c, d) PyC600, (e, f) PyC700, and (g, h) PyC800.

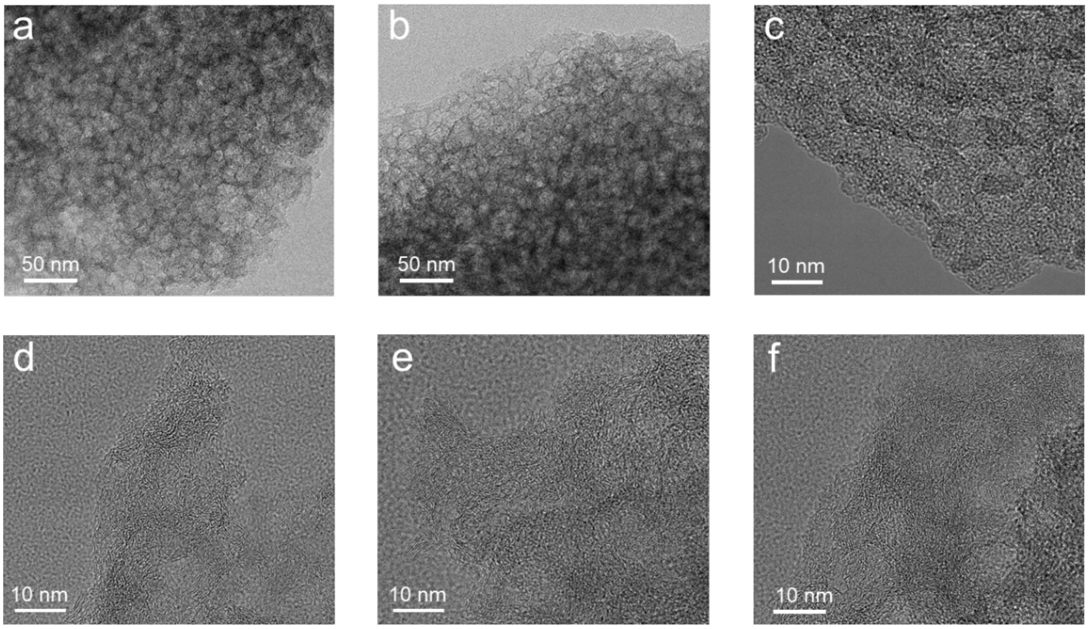

Supplementary Fig. 2. (a, b) TEM images of PyC700, and HRTEM images of (c) PyC500, (d) PyC600, (e) PyC700, and (f) PyC800.

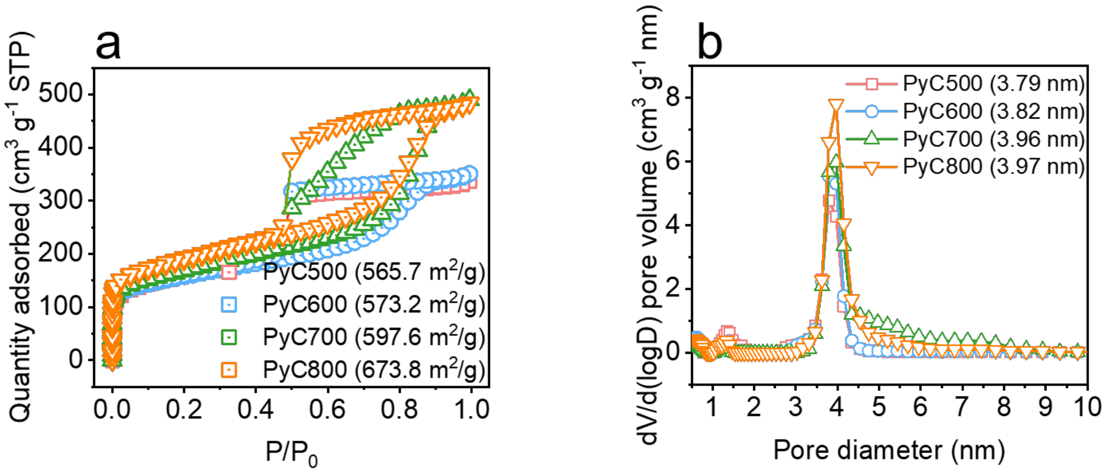

Supplementary Fig. 3. (a) N_2 adsorption-desorption isotherms and (b) pore size distribution of PyC500, PyC600, PyC700, and PyC800.

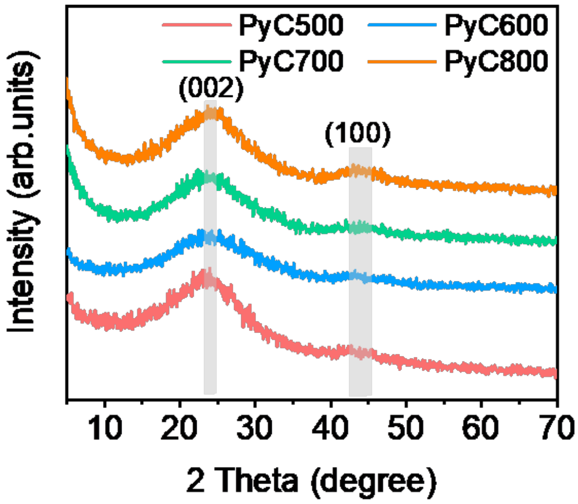

Supplementary Fig. 4. XRD patterns of the synthesized PyC500, PyC600, PyC700, and PyC800.

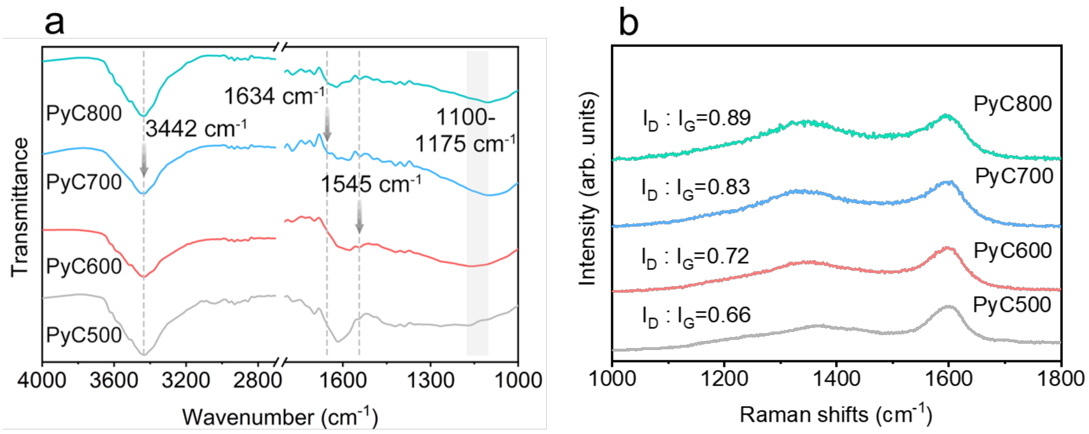

Supplementary Fig. 5. (a) FTIR and (b) Raman spectra of the PyC500, PyC600, PyC700, and PyC800.

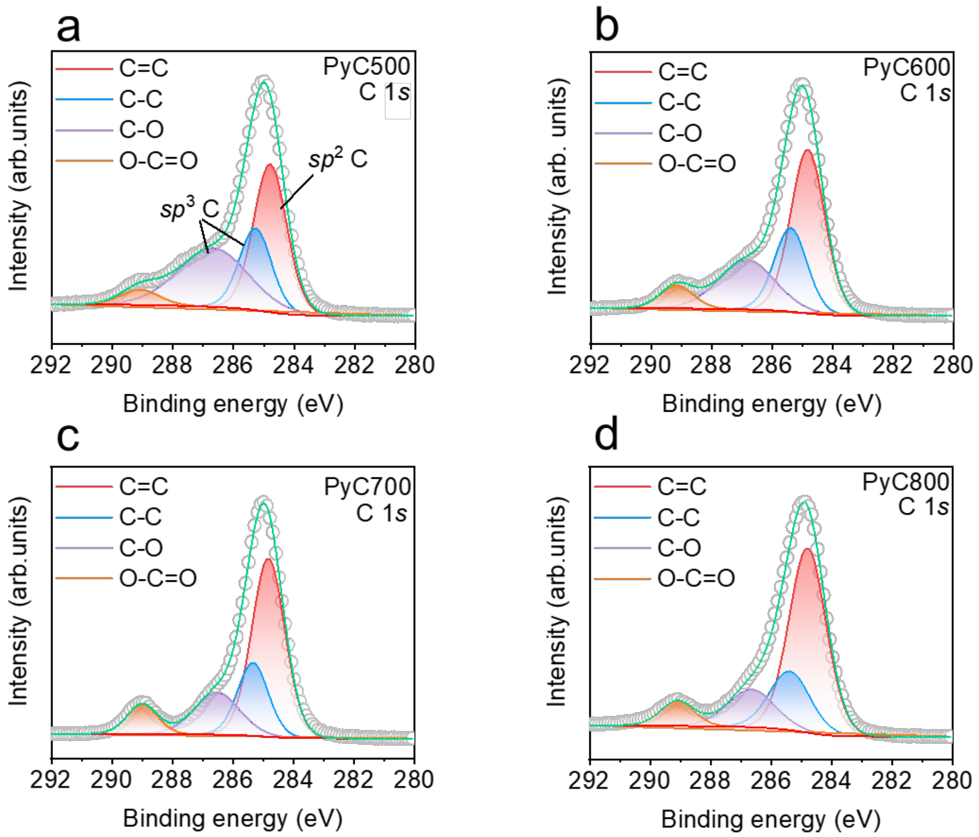

Supplementary Fig. 6. High-resolution XPS C 1*s* spectra peaks for pyrocarbon (a) PyC500, (b) PyC600, (c) PyC700, and (d) PyC800.

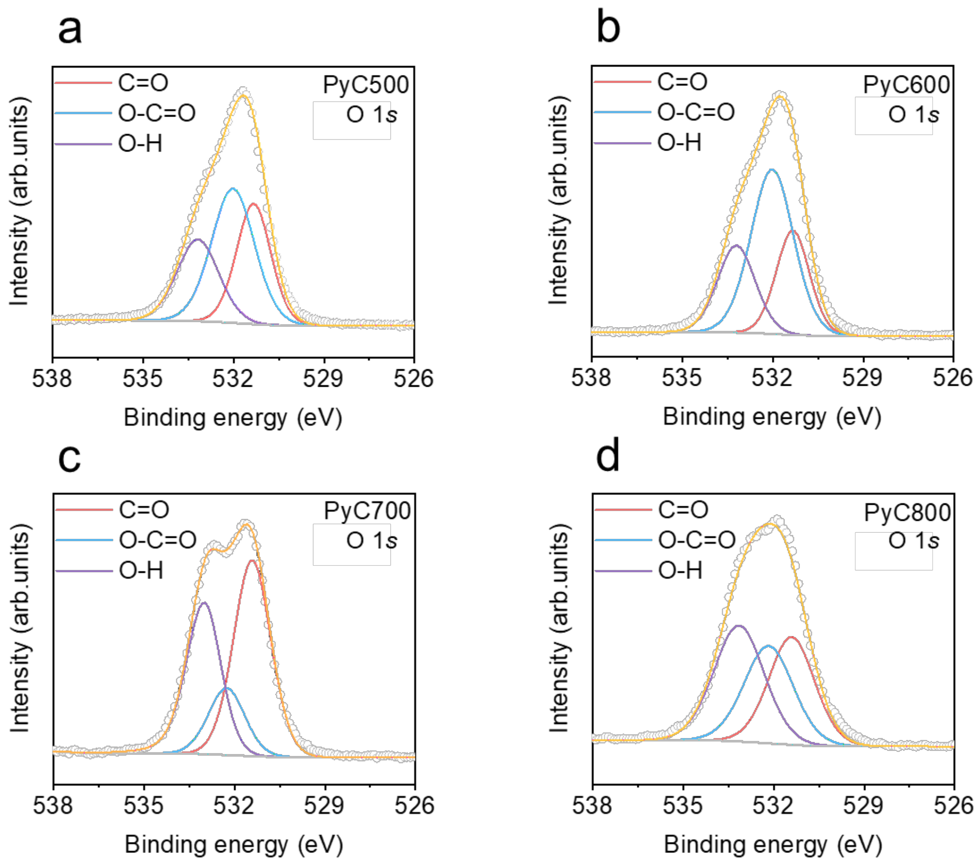

Supplementary Fig. 7. High-resolution XPS O 1*s* spectra peaks for pyrocarbon (a) PyC500, (b) PyC600, (c) PyC700, and (d) PyC800.

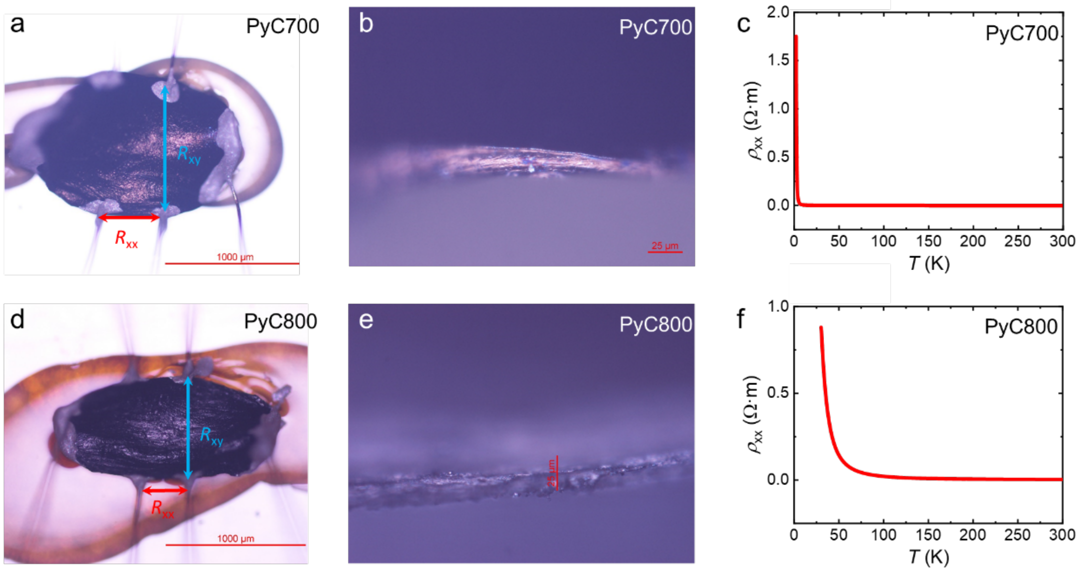

Supplementary Fig. 8. (a, b) The optical micrographs and the (c) temperature dependence of resistivity for PyC700. (d, e) The optical micrographs and the (f) temperature dependence of resistivity for PyC800. The data for PyC500 and PyC600 were omitted because of their low conductivities.

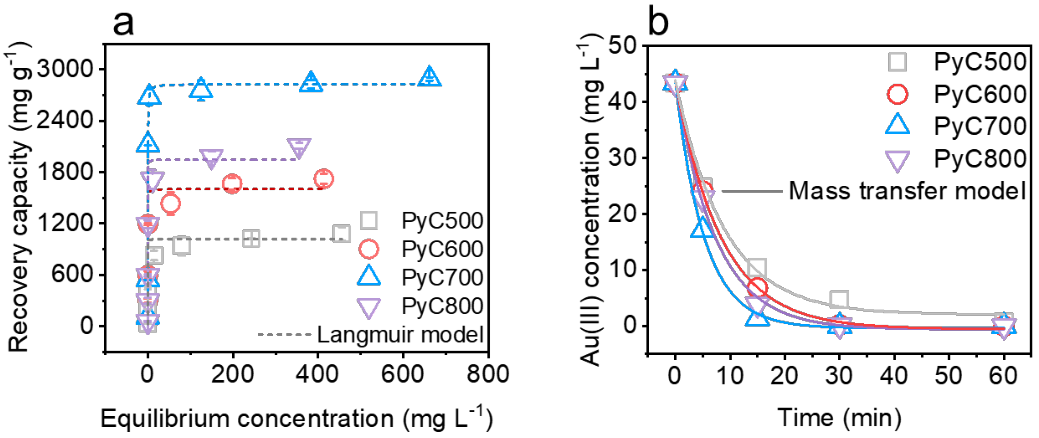

Supplementary Fig. 9. (a) Isotherms fitted by Langmuir model for Au(III) adsorption onto pyrocarbon, and (b) Kinetics fitted by mass transfer model for Au(III) adsorption onto pyrocarbon. The classical Langmuir model was employed to evaluate the theoretical gold recovery capacity of PyCs. All isotherm curves were well fitted using Langmuir model with the correlation coefficient (R^2) > 0.91 (Supplementary Table 2), indicating the effectiveness in capacity evaluation by this model. Mass transfer model was used in kinetics study to analysis the mass transfer coefficient (k_f), in which the effective area *a* in adsorption systems was provided in Supplementary Table 3. Error bars denote standard deviation of the experiments performed in triplicate.

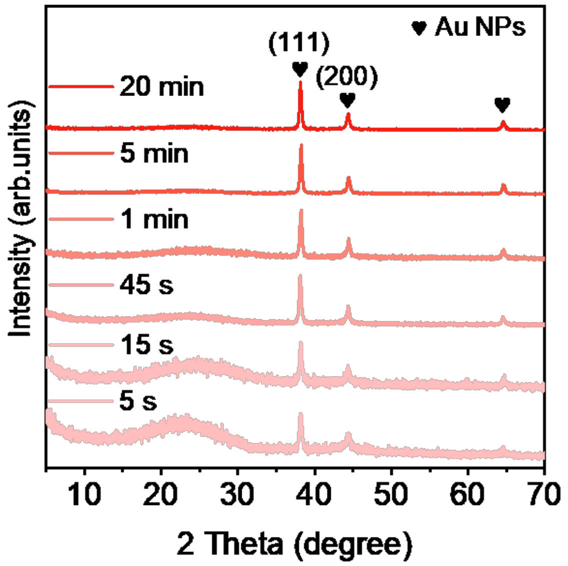

Supplementary Fig. 10. XRD patterns of the PyC700 for adsorption of Au(III) at varying reaction times.

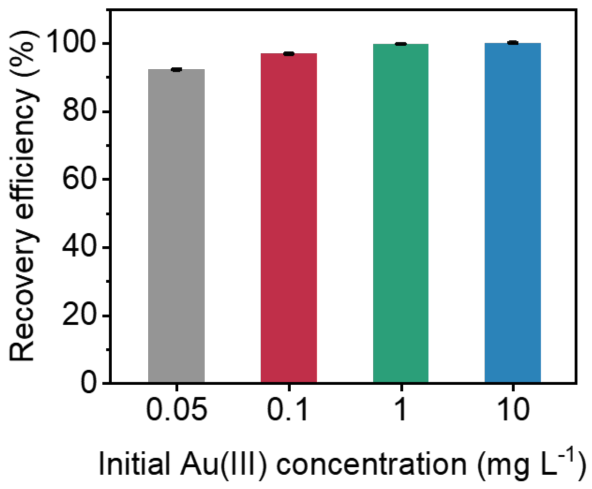

Supplementary Fig. 11. Au(III) recovery performance of PyC700 at low Au(III) concentrations. Error bars denote standard deviation of the experiments performed in triplicate.

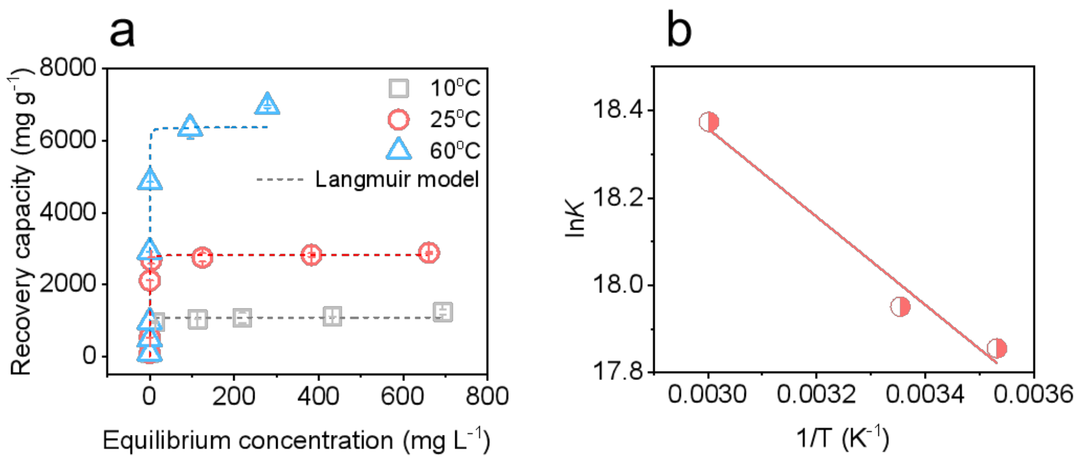

Supplementary Fig. 12. (a) Adsorption isotherms for Au(III) recovery by PyC700 at temperatures of 10°C, 25°C, and 60°C. (b) Plot of lnK versus 1/T for for Au(III) recovery by PyC700. Error bars denote standard deviation of the experiments performed in triplicate.

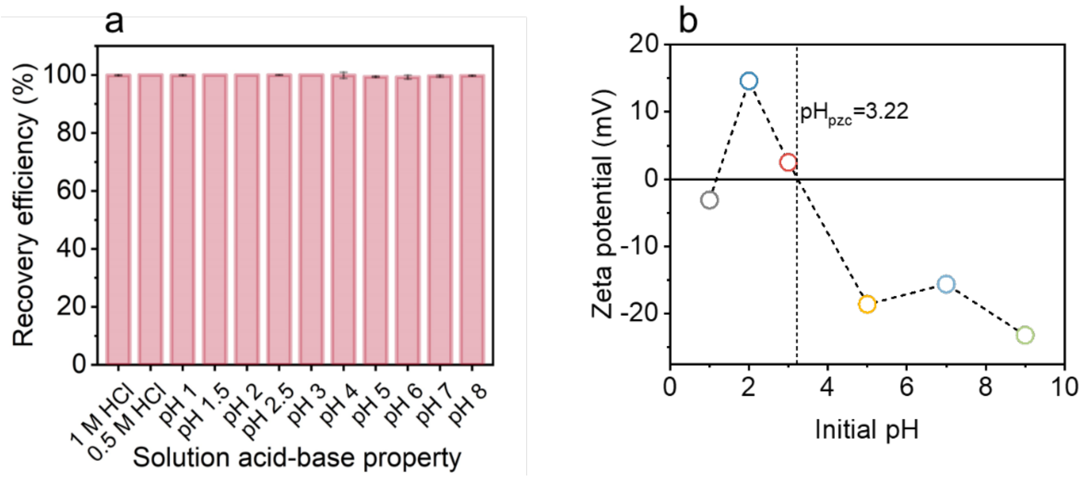

Supplementary Fig. 13. (a) Effects of solution acid-base property on Au(III) recovery efficiency (strong base environments were not considered due to their rarity in E-waste leachate). (b) Point of zero charge of the pyrocarbon in the 0.01 mol L^{-1} NaCl solution. Error bars denote standard deviation of the experiments performed in triplicate.

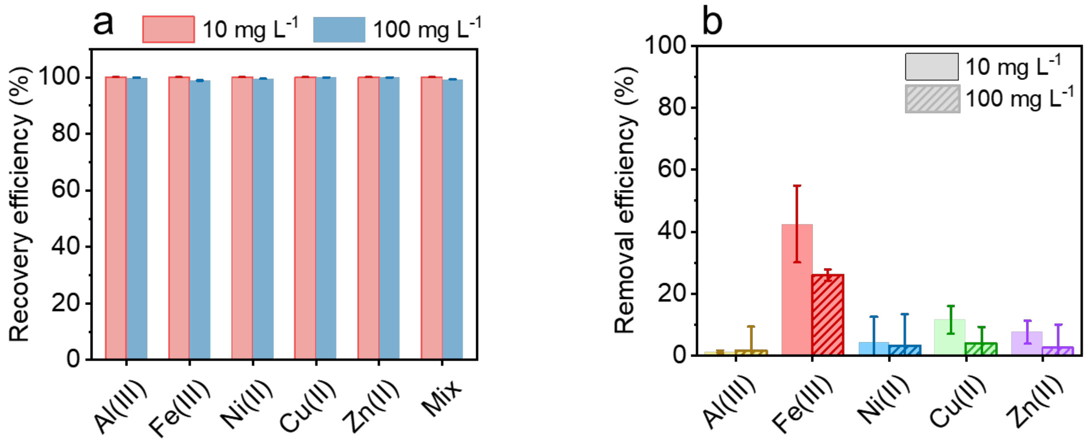

Supplementary Fig. 14. (a) Effects of competing cations on Au(III) recovery efficiency by PyC700 (initial cations concentration: 10 or 100 mg L^{-1}; the label of "Mix" means a mixture containing 5 competing cations of equal concentration)). (b) The removal efficiency of PyC700 for various competing cations. Error bars denote standard deviation of the experiments performed in triplicate.

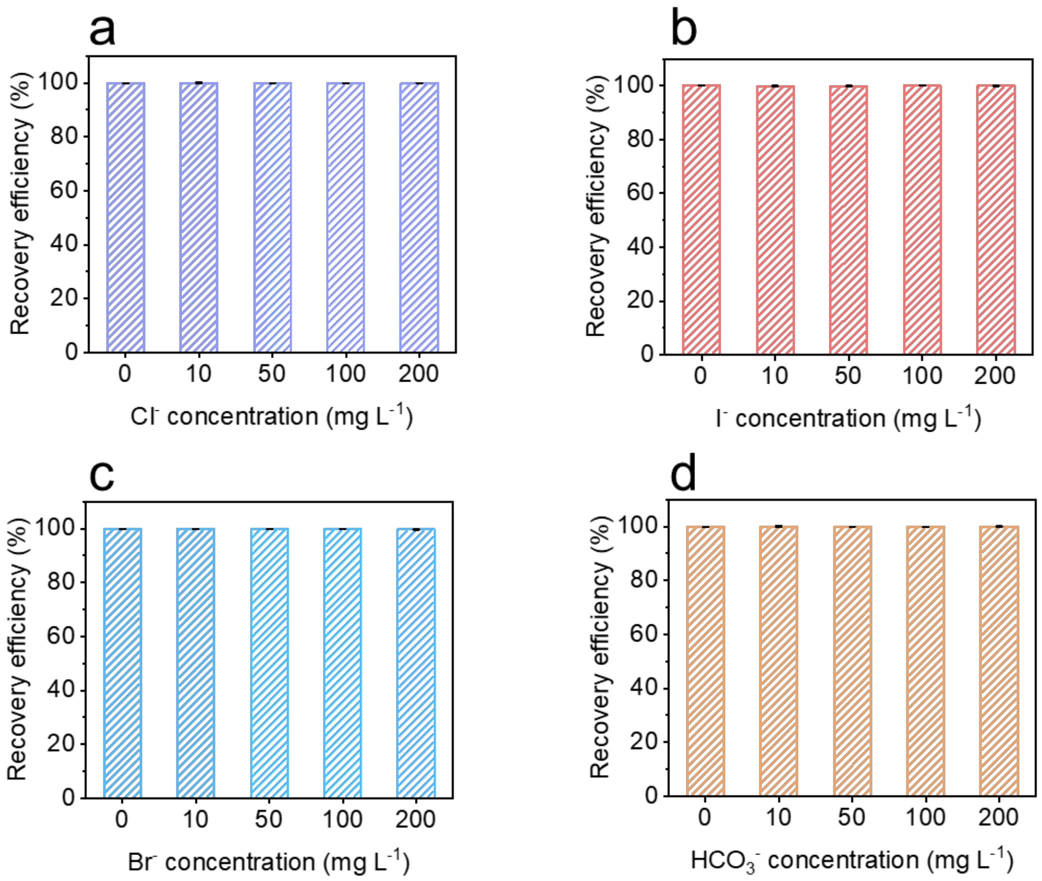

Supplementary Fig. 15. Effects of competing anions (a) Cl^-, (b) I^-, (c) Br^-, and (d) HCO_3^- on Au(III) recovery efficiency by PyC700. Error bars denote standard deviation of the experiments performed in triplicate.

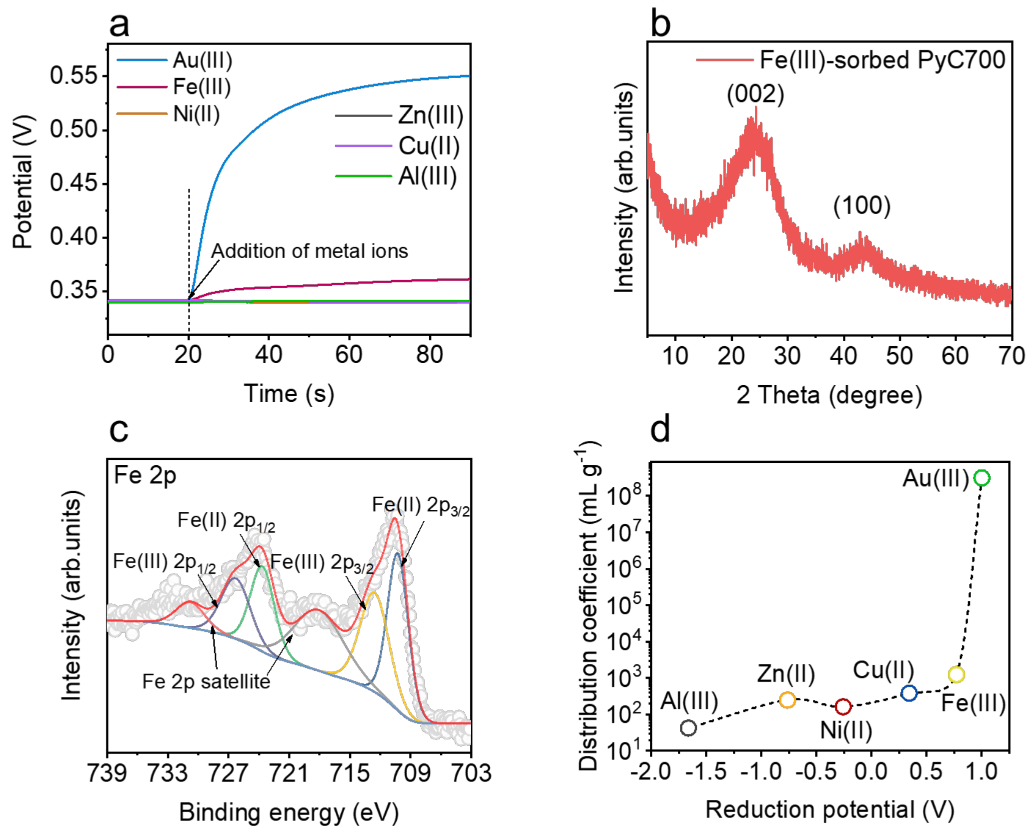

Supplementary Fig. 16. (a) The OCPT curves on the PyC700 electrodes in various competing cations solution systems. (b) XRD pattern of the PyC700 after the adsorption of Fe(III) in 500 mg L^{-1} $FeCl_3$ solution. (c) XPS analysis of Fe 2*p* in a high-resolution of Fe(III)-sorbed PyC700 sample. (d) the K_d values for various coexisting cations with different reduction potential. Minimal electrons transfer implied the redox reaction occurring between Fe(III) and PyC700 electrode, as reflected by the OPCT fluctuation. Considering higher redox potential of Fe(III)/Fe(II) ($E^0_{Fe(III)/Fe(II)}$ =+0.77 V) than Fe(III)/Fe^0 ($E^0_{Fe(III)/Fe(0)}$ =-0.037 V), the reductant can be most probably divalent Fe(II), rather than metallic Fe^0. This speculation was well manifested by XRD and XPS results in Supplementary Fig. 16b and 16c. Specifically, no characteristic peaks assigned to Fe^0 appeared in the XRD pattern of the PyC700 after reaction in $FeCl_3$ solution. And the deconvoluted XPS Fe 2*p* spectra indicated the copresence of Fe(III) and Fe(II) on the Fe(III)-sorbed PyC700. These results identified the partially reduction of Fe(III) to Fe(II) by PyC700; however, it exhibited no interference on the gold recovery performance, mainly due to high redox potential of Au(III)/Au^0 ($E^0_{Au(III)/Au(0)}$ =+1.002 V) and the sufficient reductive electron supply.

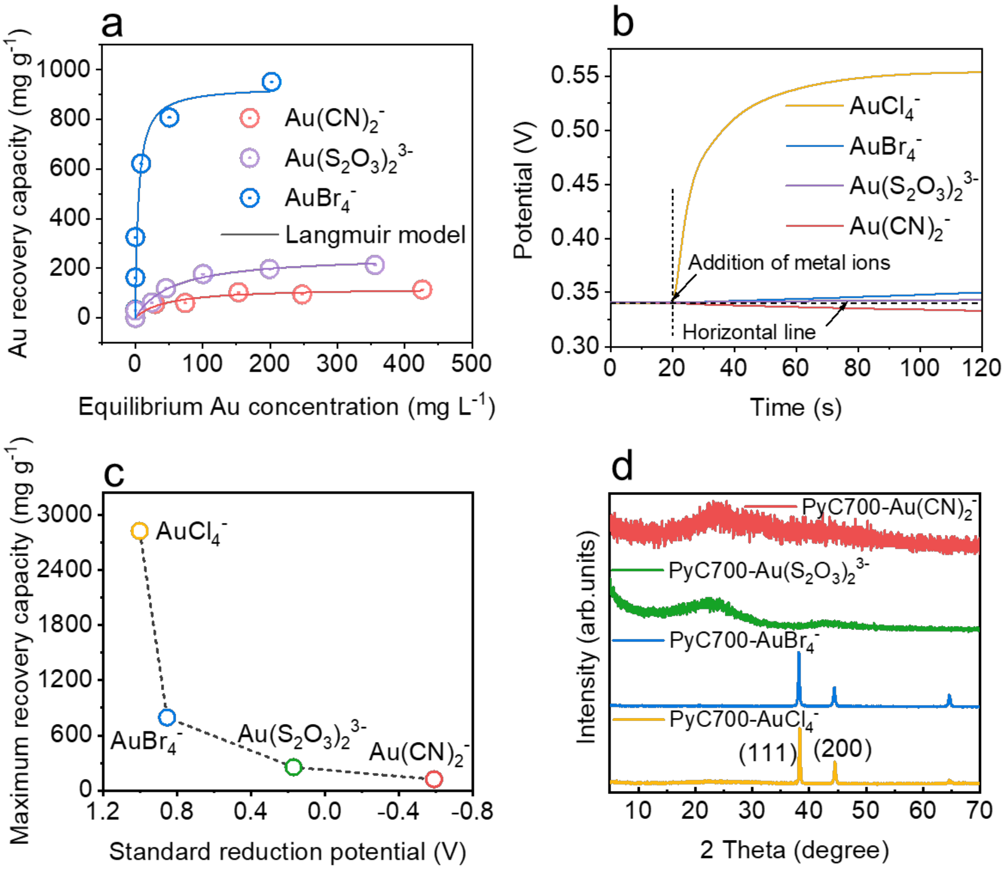

Supplementary Fig. 17. (a) Adsorption isotherms for $Au(CN)_2^-$, $Au(S_2O_3)_2^{3-}$, and $AuBr_4^-$ adsorption onto PyC700, (b) OCPT curves on the PyC700 electrodes in various Au(III) species solution systems. (c) maximum recovery capacity for various gold complexes with different reduction potential (E_0). (d) XRD patterns of the PyC700 after the gold sorption in 500 mg L^{-1} $AuCl_4^-$, $AuBr_4^-$, $Au(S_2O_3)_2^{3-}$, and $Au(CN)_2^-$ solution, respectively.

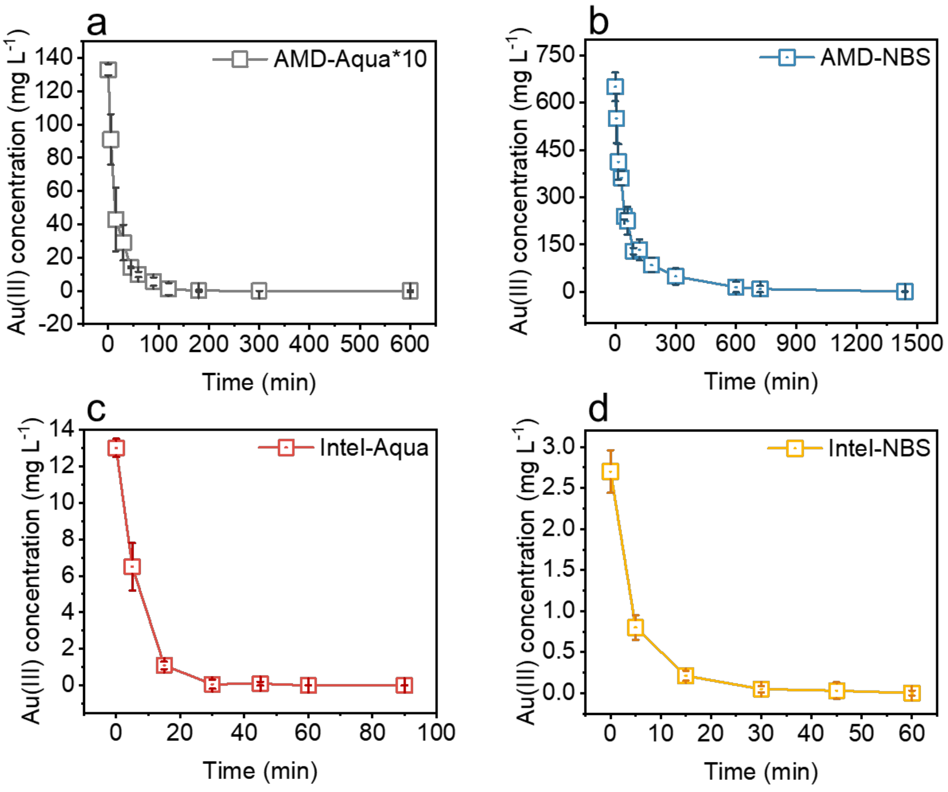

Supplementary Fig. 18. Kinetics curves for the Au(III) adsorption on PyC700 in four real CPUs leaching solutions of (a) AMD-Aqua*10, (b) AMD-NBS, (c) Intel-Aqua, and (d) Intel-NBS. PyC700 exhibits rapid adsorption kinetics at both high and relatively low concentration ranges. It reaches adsorption equilibrium within ~180 and 600 min at high Au(III) concentration of 133 and 650 mg L^{-1} in AMD-Aqua*10 and AMD-NBS leachates, respectively. Meanwhile, over than 98.0% of Au(III) can be successfully recovered from Intel-Aqua and Intel-NBS leachates within 30 min. The kinetics results verified rapid adsorption capability of the PyC700 in real E-waste leachates, showcasing its high prospect in practical adsorption equipment (e.g., mixed reactors or fixed bed column). Error bars denote standard deviation of the experiments performed in triplicate.

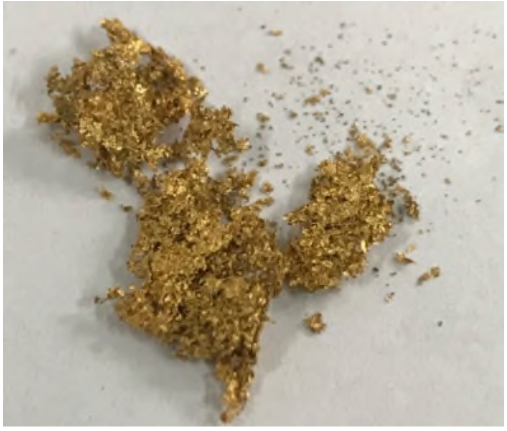

Supplementary Fig. 19. Optical photograph of the obtained gold foil from spent PyC700 in actual E-waste leachate.

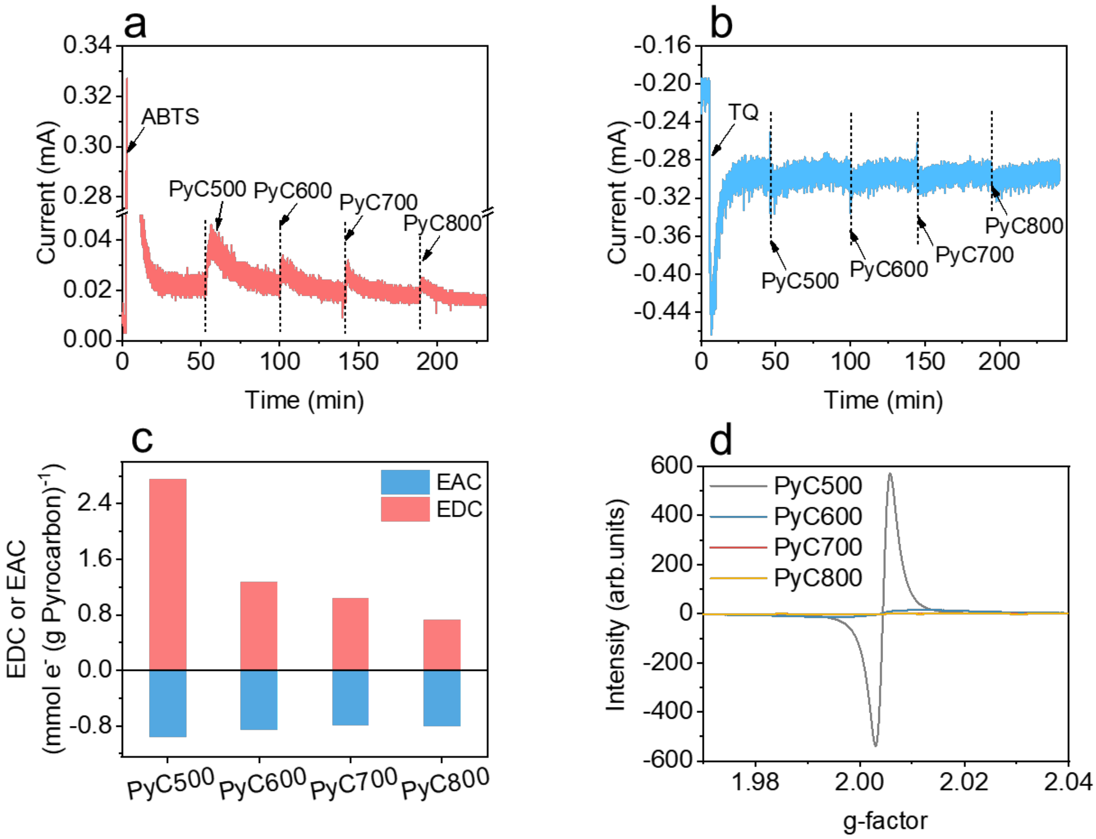

Supplementary Fig. 20. (a) Reductive and (b) oxidative current analyzed by mediated electrochemical oxidation/reduction (red/blue curve) and (c) electron-donating/accepting capacities (EDC/EAC, red/blue column) of PyCs. (d) EPR spectra of PyCs. The EAC/EDC of pyrocarbon illustrated the capability of storing the excess electrons and releasing electrons to the electron acceptors. Accordingly, the reversible ability of pyrocarbon to release/accept electrons

(corresponding to EDC and EAC, respectively) could be attributed to the reversible transformation of hydroquinone/quinone groups, respectively. We also found a close correlation between EDC and the amount of phenolic hydroxyl (-OH) (determined using Boehm titration). The measured EDC/EAC values of the pyrocarbon samples were well in line with those in previously reported studies.[5, 15] However, both the amount of phenolic -OH (1.0 to 3.1 mmol g^{-1}) and EDC (0.73 to 2.76 mmol g^{-1}) of PyCs was significantly lower than the electron amount required to reduce gold ions (15.6 to 96.9 mmol g^{-1}), which excludes the role of intrinsic phenolic -OH groups in contributing electrons during Au(III) recovery. Singlet EPR signals were observed for PyC500 with g-factor at 2.0044, which suggested the formation of oxygen-centered persistent free radicals (e.g., C-O•) during pyrolysis at 500ºC. However, only weak signals were observed in the EPR spectra of PyC600, PyC700, and PyC800, indicating the absence or limited presence of persistent free radicals. Therefore, the EPR results could exclude the reductive contribution of persistent free radicals to the gold recovery by pyrocarbon.

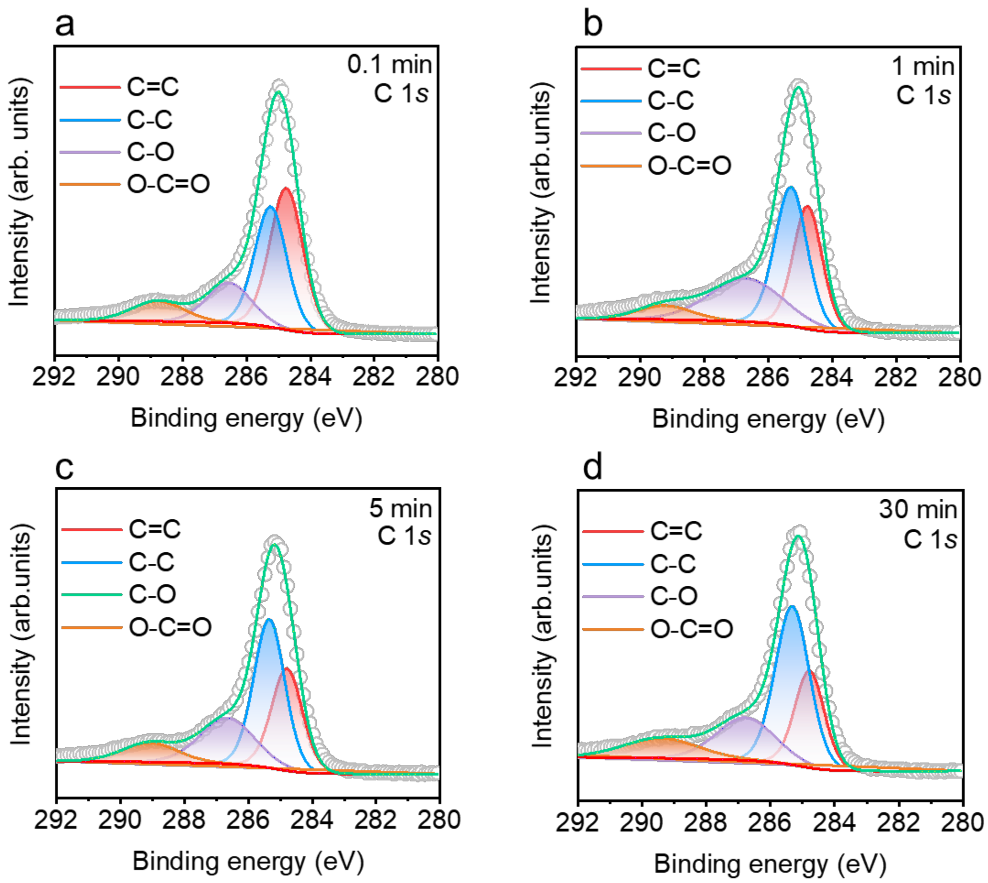

Supplementary Fig. 21. High-resolution C 1*s* XPS spectra of PyC700 loading Au(III) with reaction time of (a) 0.1 min, (b) 1 min, (c) 5 min, and (d) 30 min.

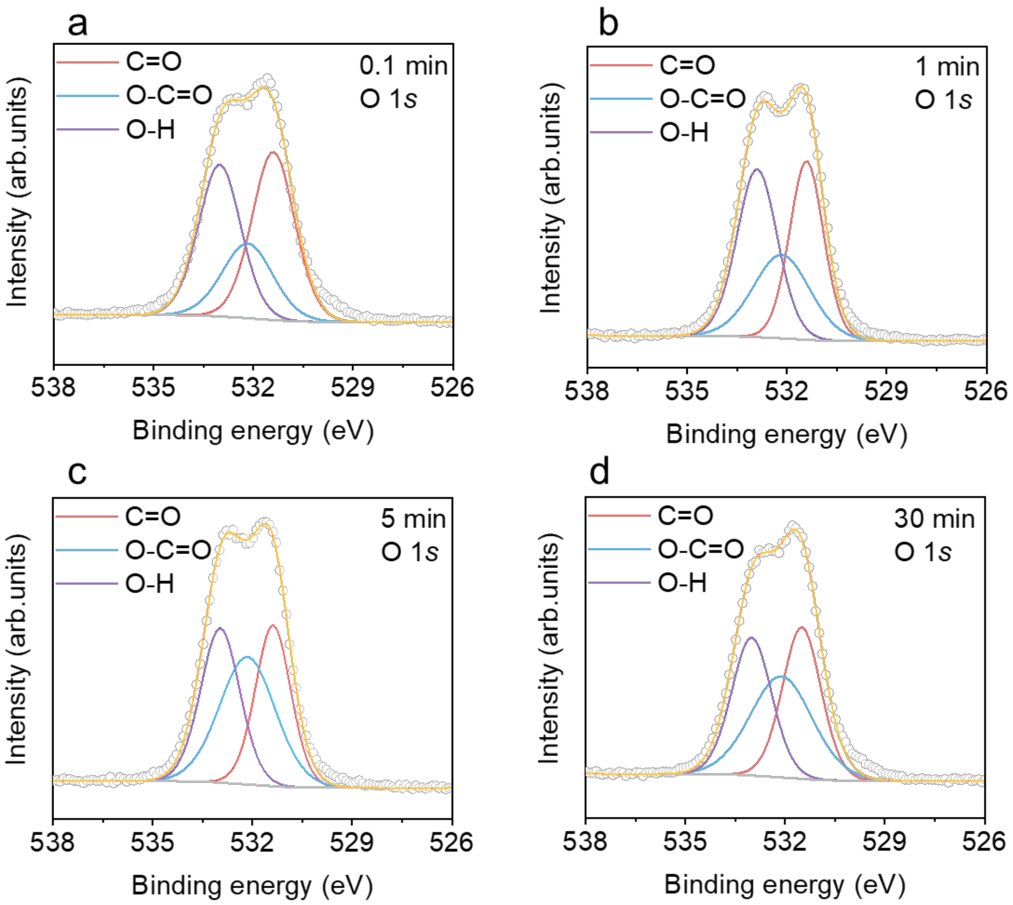

Supplementary Fig. 22. High-resolution O 1*s* XPS spectra of PyC700 loading Au(III) with reaction time of (a) 0.1 min, (b) 1 min, (c) 5 min, and (d) 30 min.

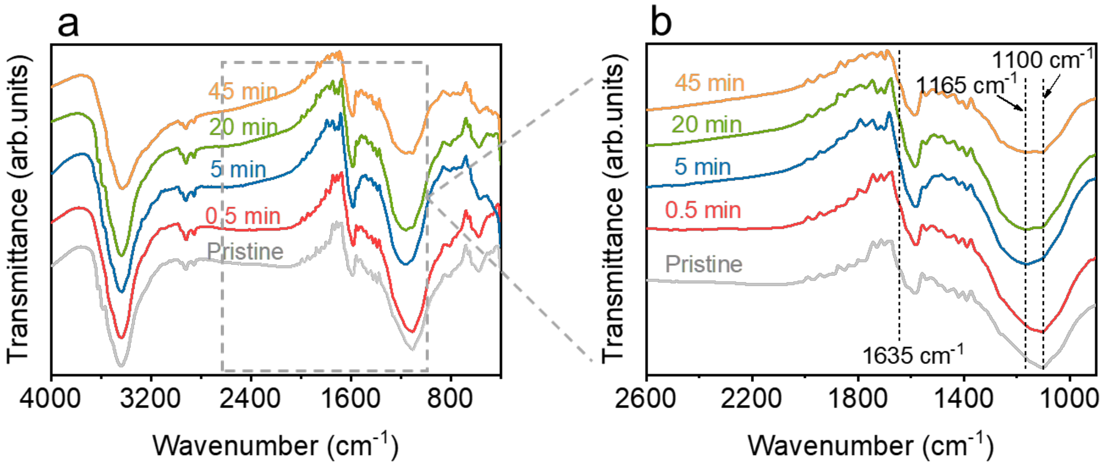

Supplementary Fig. 23. FTIR spectra in (a) full spectrum (4000 to 400 cm^{-1}) and (b) amplified spectrum (2600 to 900 cm^{-1}) of Au(III) adsorption by PyC700 at varying reaction time (0 to 45 min).

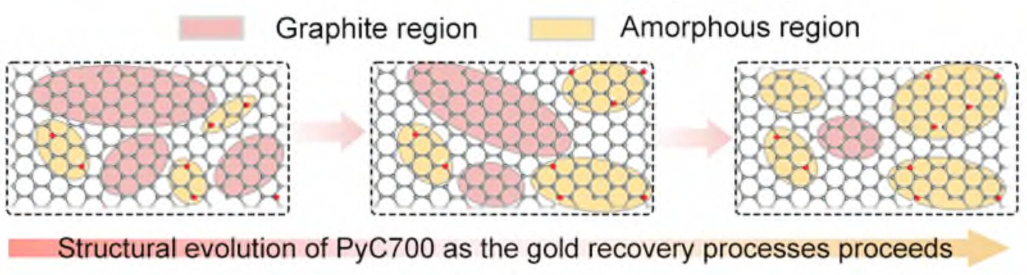

Supplementary Fig. 24. Schematic diagram of structure evolution of PyC700 during the gold recovery process.

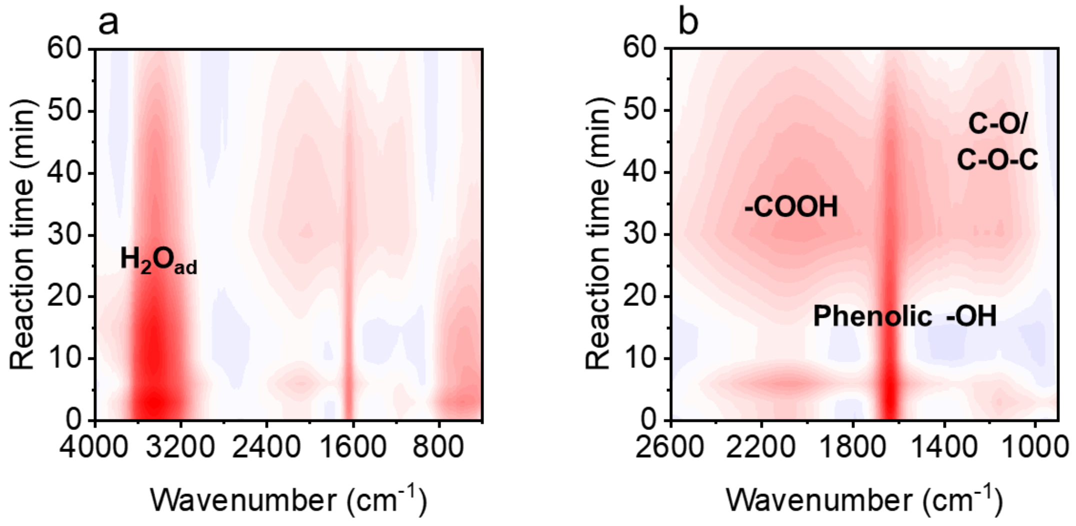

Supplementary Fig. 25. (a, b) Heat mappings of the in situ FTIR spectra for the reaction of PyC700 in Au(III) solution.

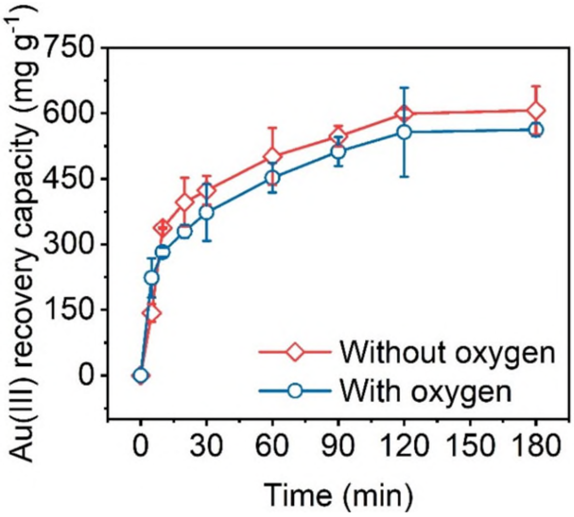

Supplementary Fig. 26. Au(III) adsorption experiments by PyC700 performed in the presence or absence of oxygen. Prior to test in the absence of oxygen, the dissolved oxygen was removed by a continuous flow of argon gas through the boiled water. Error bars denote standard deviation of the experiments performed in triplicate.

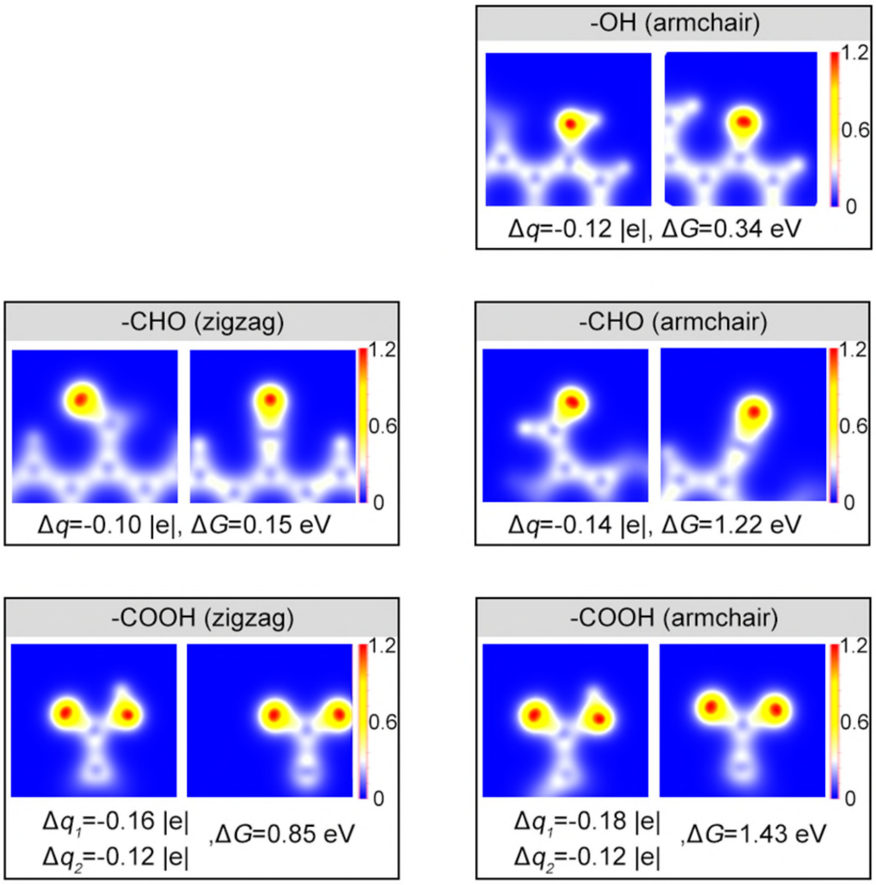

Supplementary Fig. 27. Electron localization function slice mappings of hydroxyl, aldehyde, carboxyl groups in zigzag-edged and armchair-edged graphene structures.

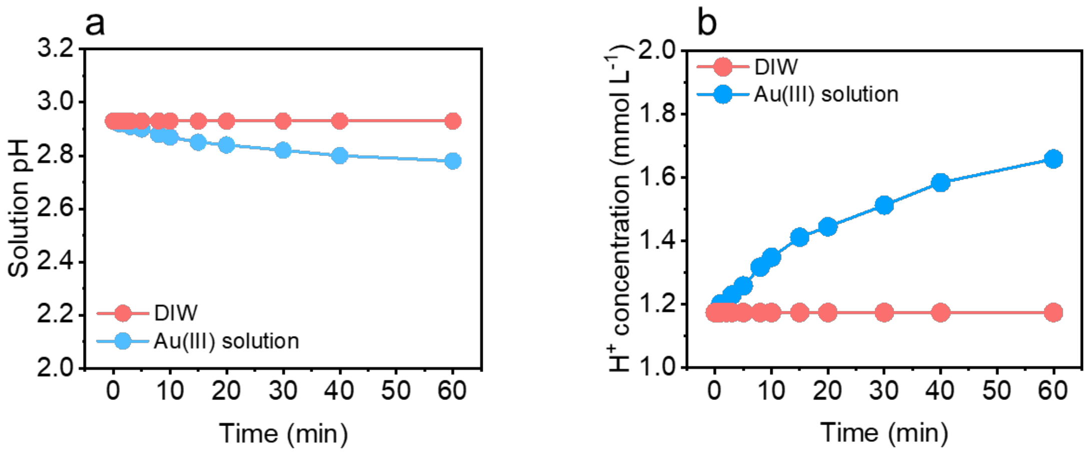

Supplementary Fig. 28. (a) Solution pH evolution and (b) H^+ concentration change diagrams for the addition of PyC700 into DIW and 200 mg L^{-1} Au(III) solution (initial pH of the solutions were pre-adjusted to 2.93). Due to water dissociation processes and redox transformations of phenolic -OH groups, a number of protons are released into solution systems, which was proved by monitoring the solution pH change during gold recovery by PyC700.

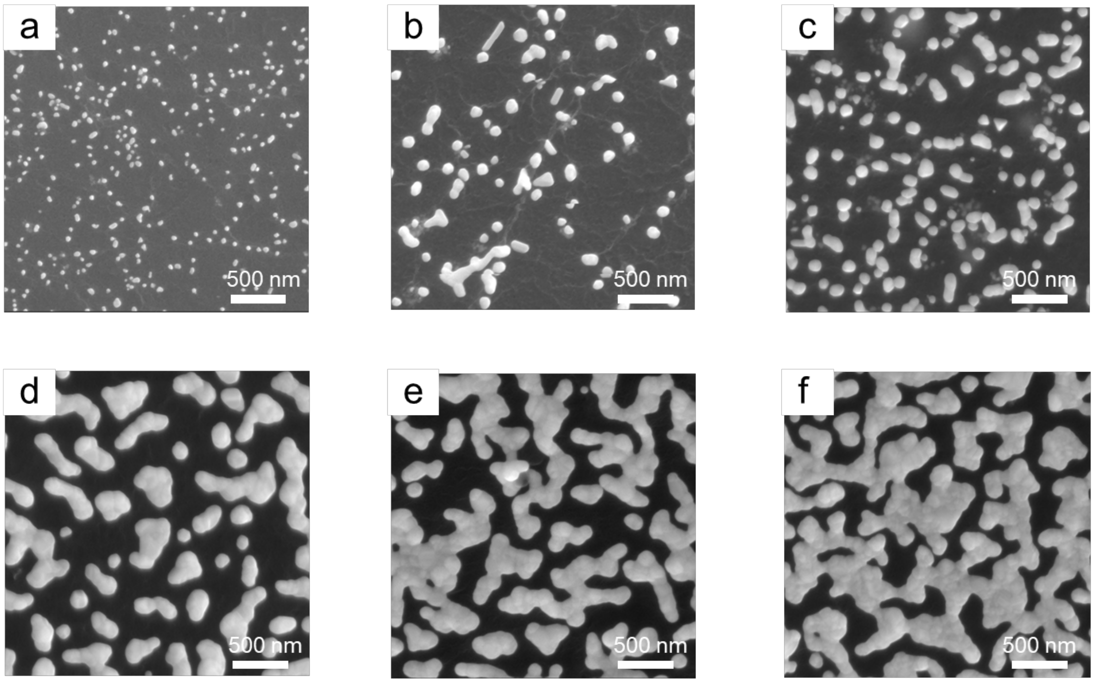

Supplementary Fig. 29. SEM images of the PyC700 adsorption in Au(III) solution for varying reaction times (a) 0.1 min, (b) 0.25 min, (c) 1 min, (d) 5 min, (e) 20 min, (f) 30 min.

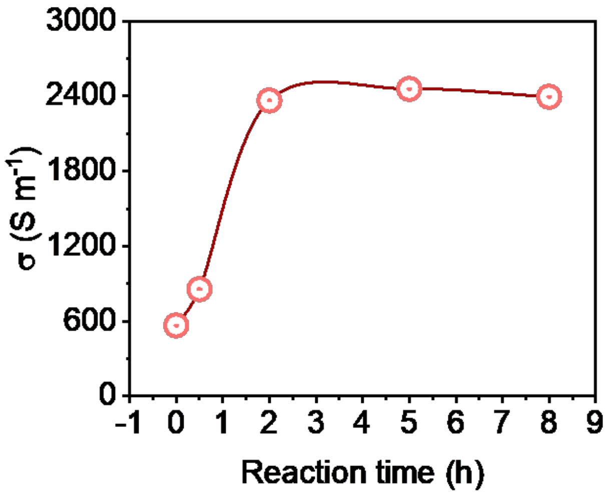

Supplementary Fig. 30. The electronic conductivity of the PyC700 sample that adsorbed Au(III) for varying reaction times. After reaction in 500 mg L^{-1} $AuCl_4^-$ solution for certain period, the PyC700 samples were freezing-dried, and their conductivities were measured via four-point probe method with resistivity tester (Malvern Mastersizer 2000). Based on the XRD and SEM results (Supplementary Fig. 10 and 29), we observed a consecutive reduction of Au(III) salt to elemental gold during the adsorption processes, followed by the formation of nanoscale Au seeds. These Au seeds exhibit a high surface free energy, rendering them unstable in a 'boiling state' that promotes the consumption of Au(III) salt and facilitates electron transfer for the aggregation of Au^0 NPs. Our findings in Supplementary Fig. 30 further demonstrate a significant enhancement in PyC700 conductivity due to the aggregation of Au^0 NPs on its surfaces during the initial gold recovery stage. Therefore, it is hypothesized that both enhanced reactivity and electron transfer contribute to efficient Au(III) recovery on the PyC700 sorbent, which can be referred to as autocatalytic surface growth based on previous studies.[16, 17]

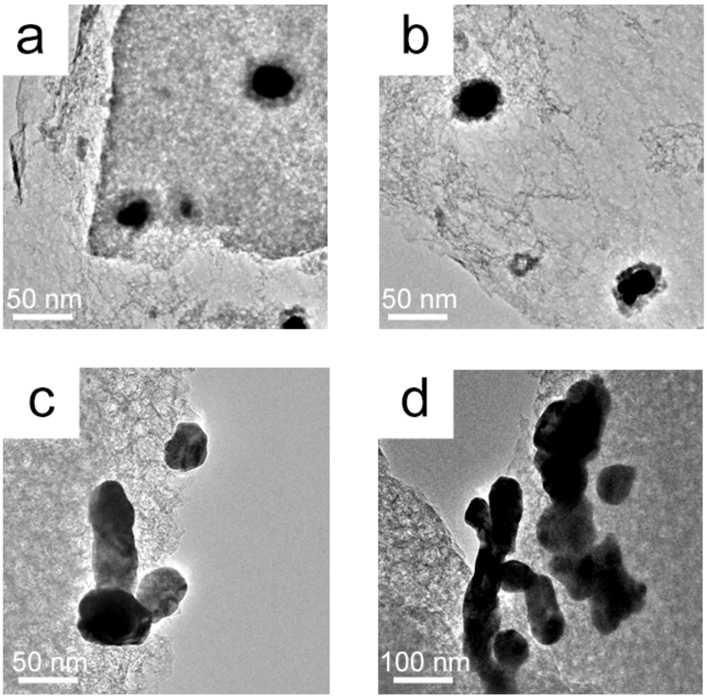

Supplementary Fig. 31. TEM images of the PyC700 adsorption in Au(III) solution for (a, b) 0.1 min, (c) 0.25 min, and (d) 1 min.

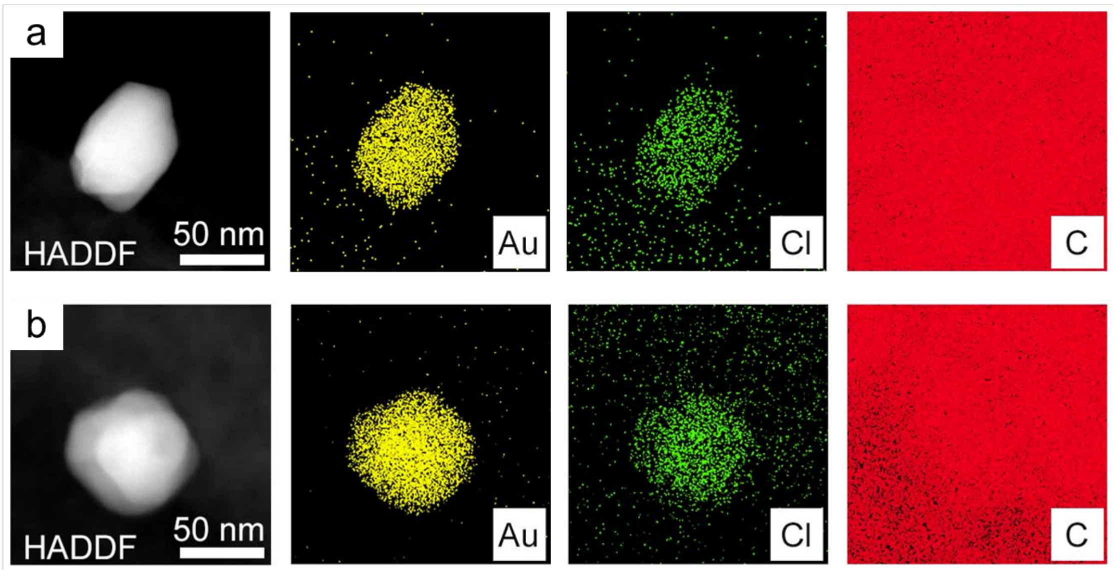

Supplementary Fig. 32. HAADF-STEM images of the PyC700 adsorption in Au(III) solution for (a) 0.25 min and (b) 1 min. EDS mapping images of Au, Cl, and C elements were provided along with the HAADF-STEM images.

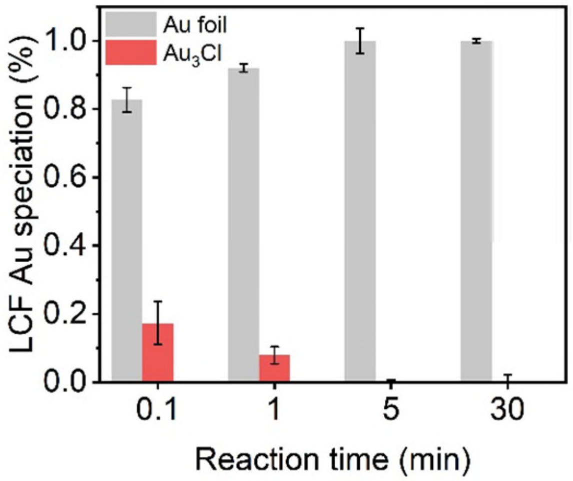

Supplementary Fig. 33. LCF of the Au speciation from the Au L_3-edge XANES spectra of the PyC700 PyC700 treated in Au(III) solution for varying reaction times (0.1 to 30 min). Only Au foil and Au_3Cl standards were used in LCF analysis due to low valence states of Au in Au-loaded PyC700 compared with Au(III) in $Au(OH)_3$.

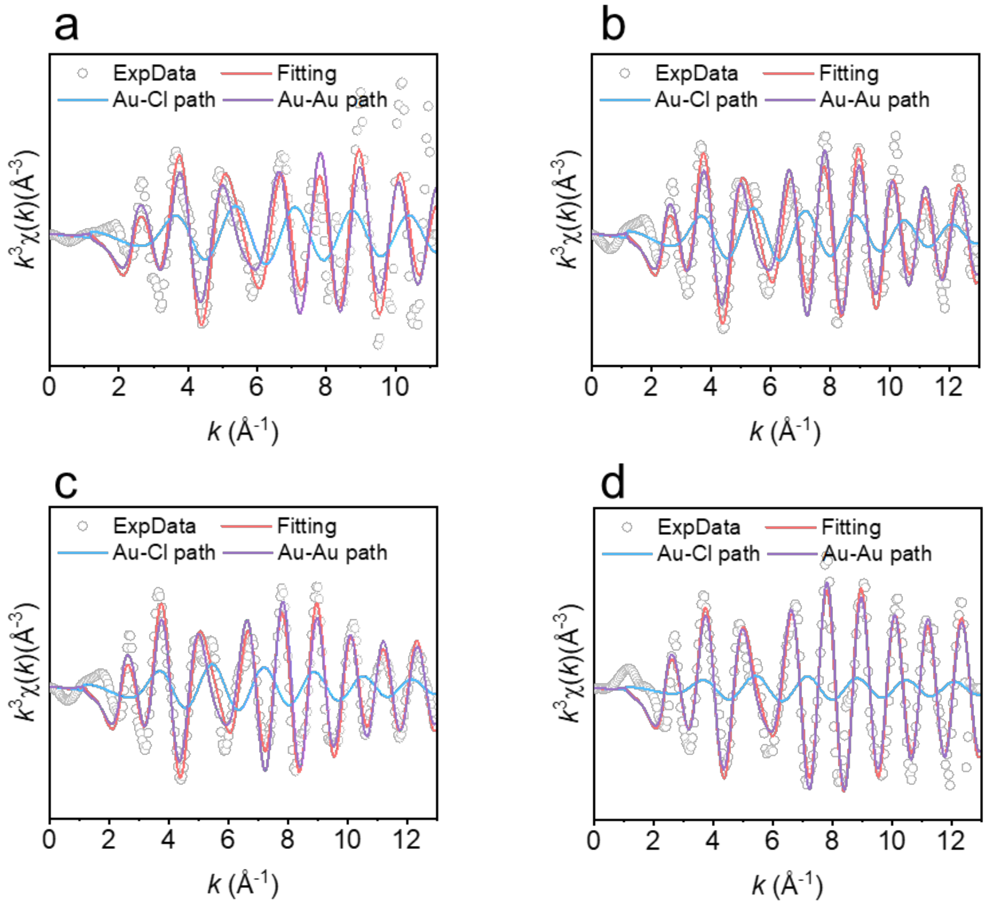

Supplementary Fig. 34. k-space EXAFS spectra with fits of Au-Cl and Au-Au scattering paths for PyC700 treated in Au(III) solution for (a) 0.1, (b) 1, (c) 5, and (d) 30 min.

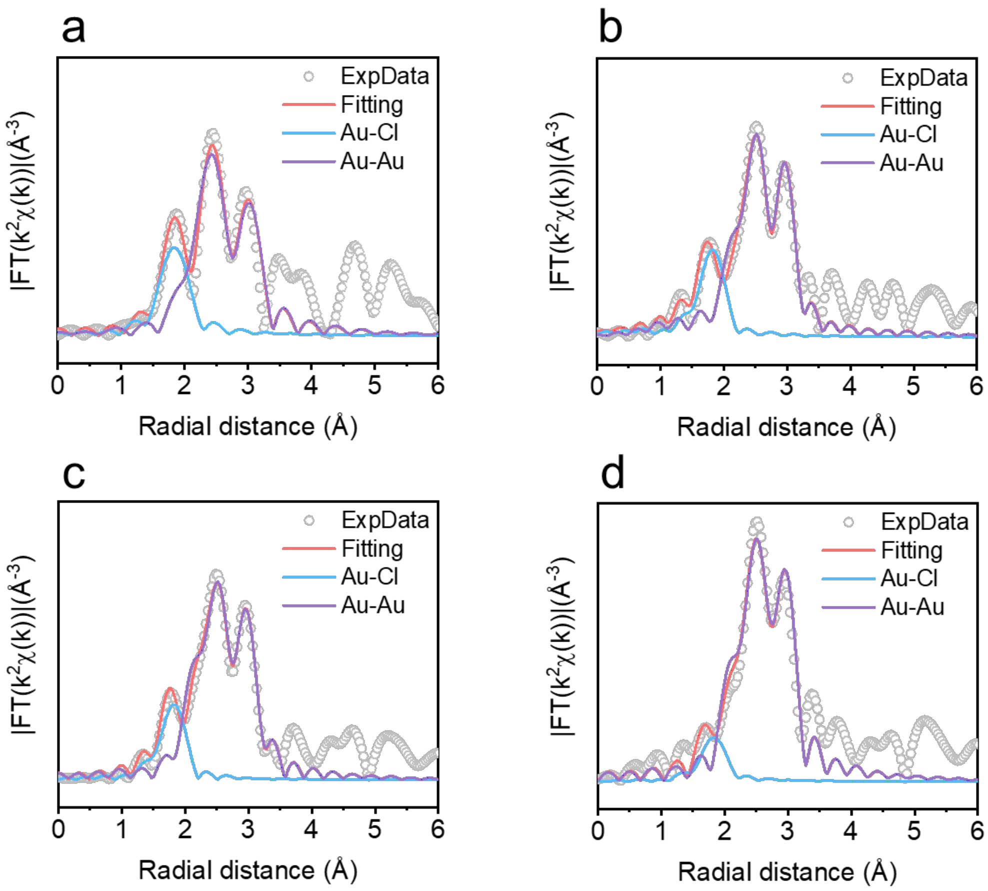

Supplementary Fig. 35. Fourier transform of the k^2-weighted EXAFS spectra with fits of Au-Cl and Au-Au scattering paths for PyC700 treated in Au(III) solution for (a) 0.1, (b) 1, (c) 5, and (d) 30 min.

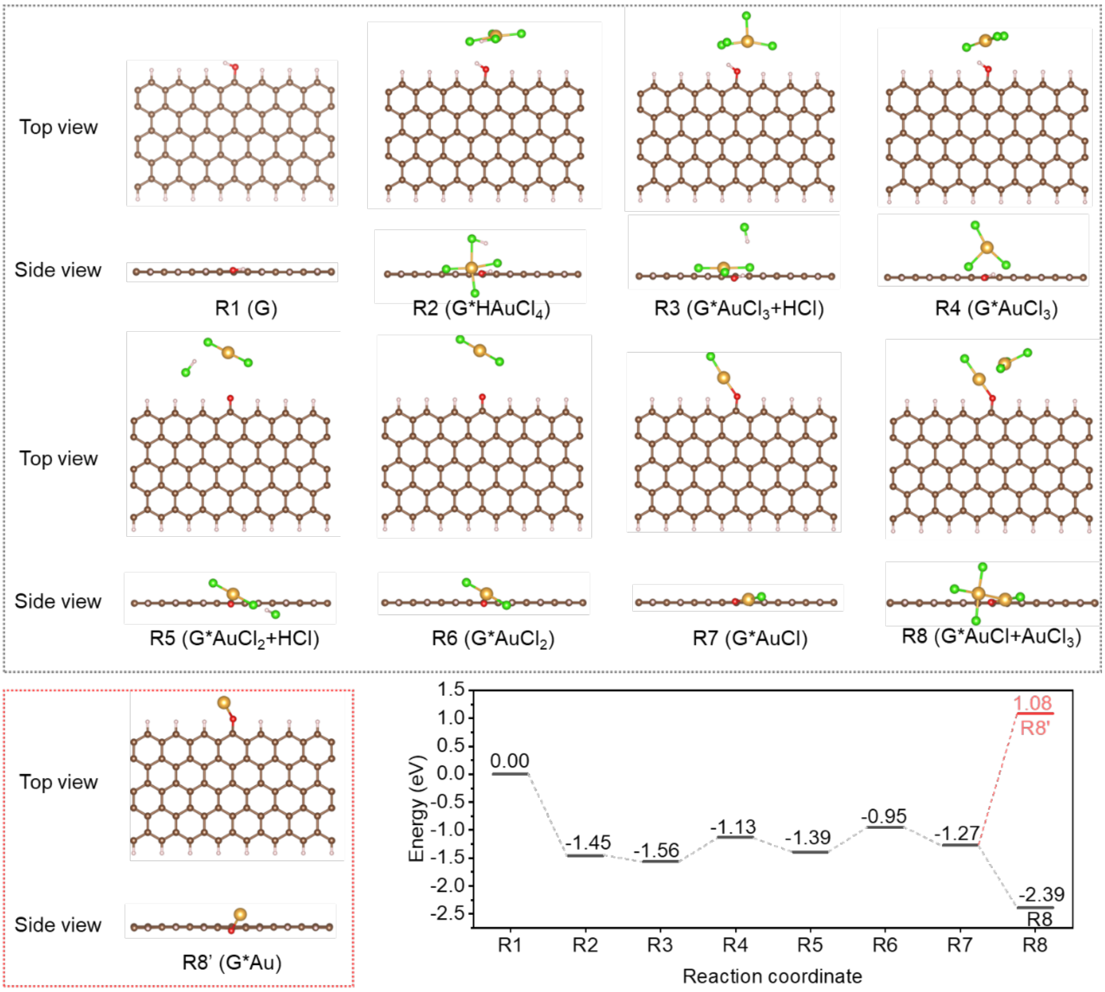

Supplementary Fig. 36. Schematic diagram and calculated energy diagram for the Au(III) ($HAuCl_4$) adsorption and reduction on PyC700 from reaction coordinate 1 to 8 (R1 to R8) (R8' represents another possible coordination configuration after R7, however, it exhibits a higher reaction energy barrier compared to the optimal R8 we have inferred).

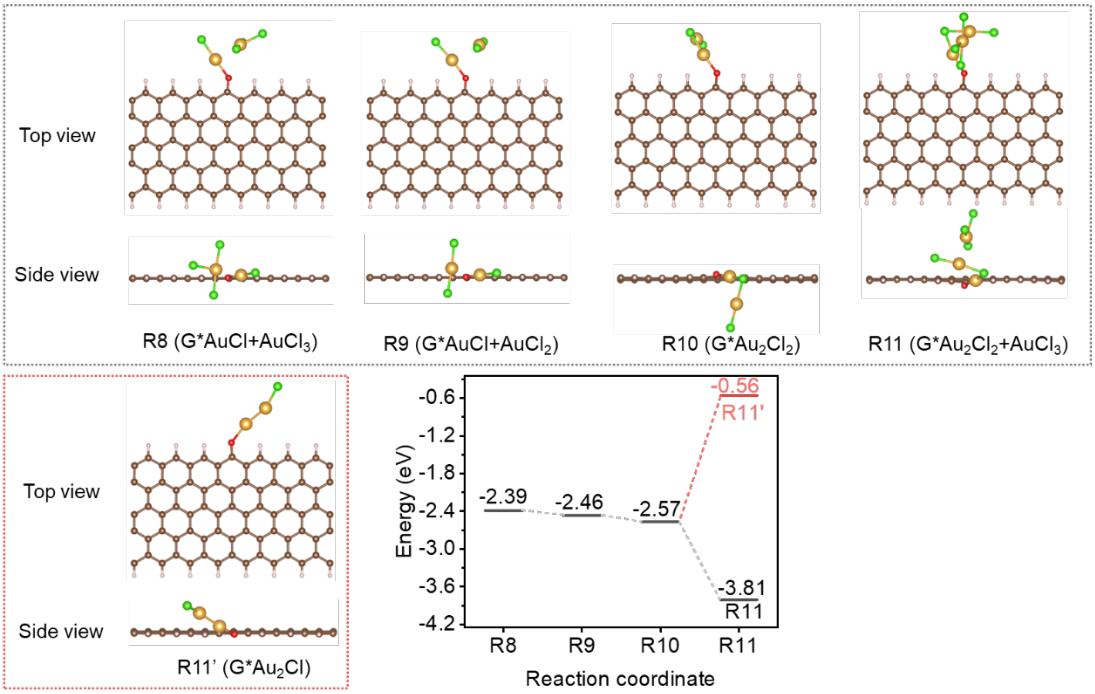

Supplementary Fig. 37. Schematic diagram and calculated energy diagram for the Au(III) ($AuCl_3$) adsorption and reduction on PyC700 from reaction coordinate 8 to 11 (R8 to R11) (R11' represents another possible coordination configuration after R10, however, it exhibits a higher reaction energy barrier compared to the optimal R11 we have inferred).

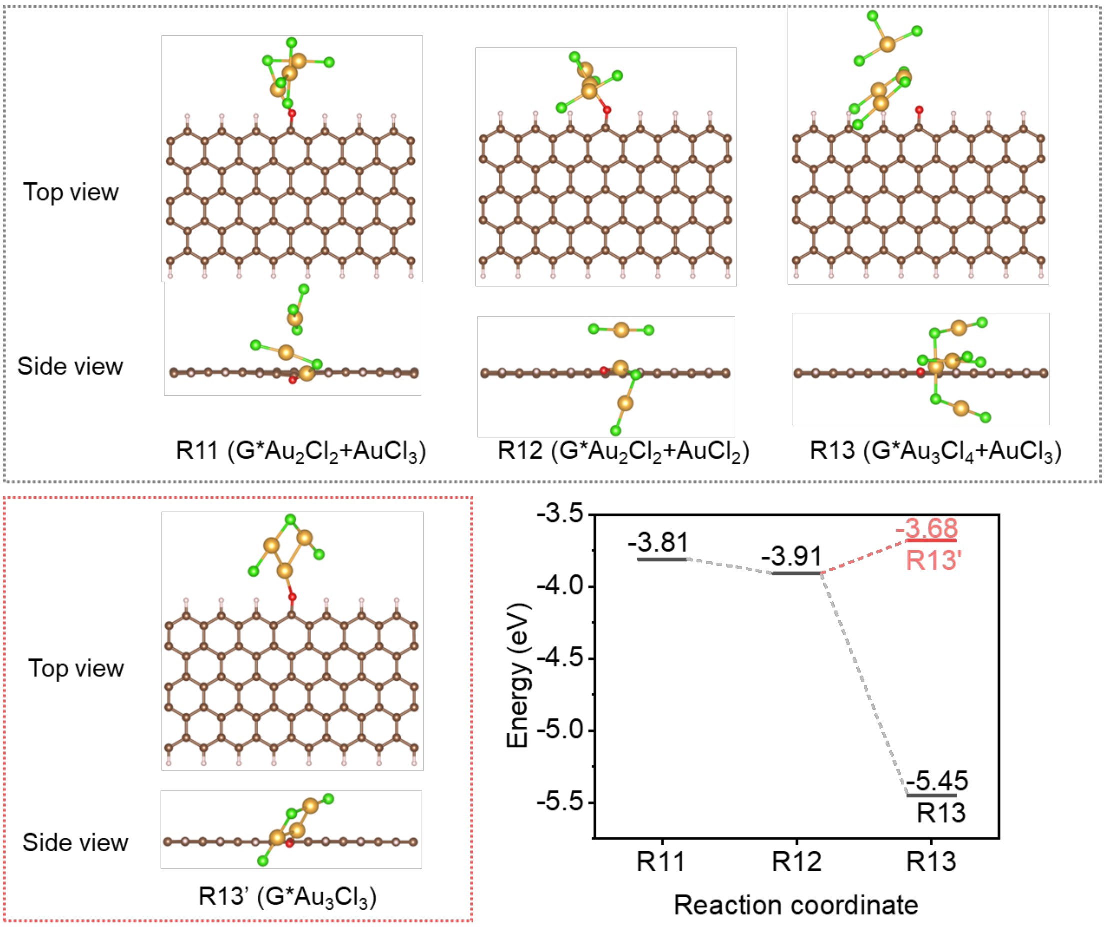

Supplementary Fig. 38. Schematic diagram and calculated energy diagram for the Au(III) ($AuCl_3$) adsorption and reduction on PyC700 from reaction coordinate 11 to 13 (R11 to R13) (R13' represents another possible coordination configuration after R12, however, it exhibits a higher reaction energy barrier compared to the optimal R13 we have inferred).

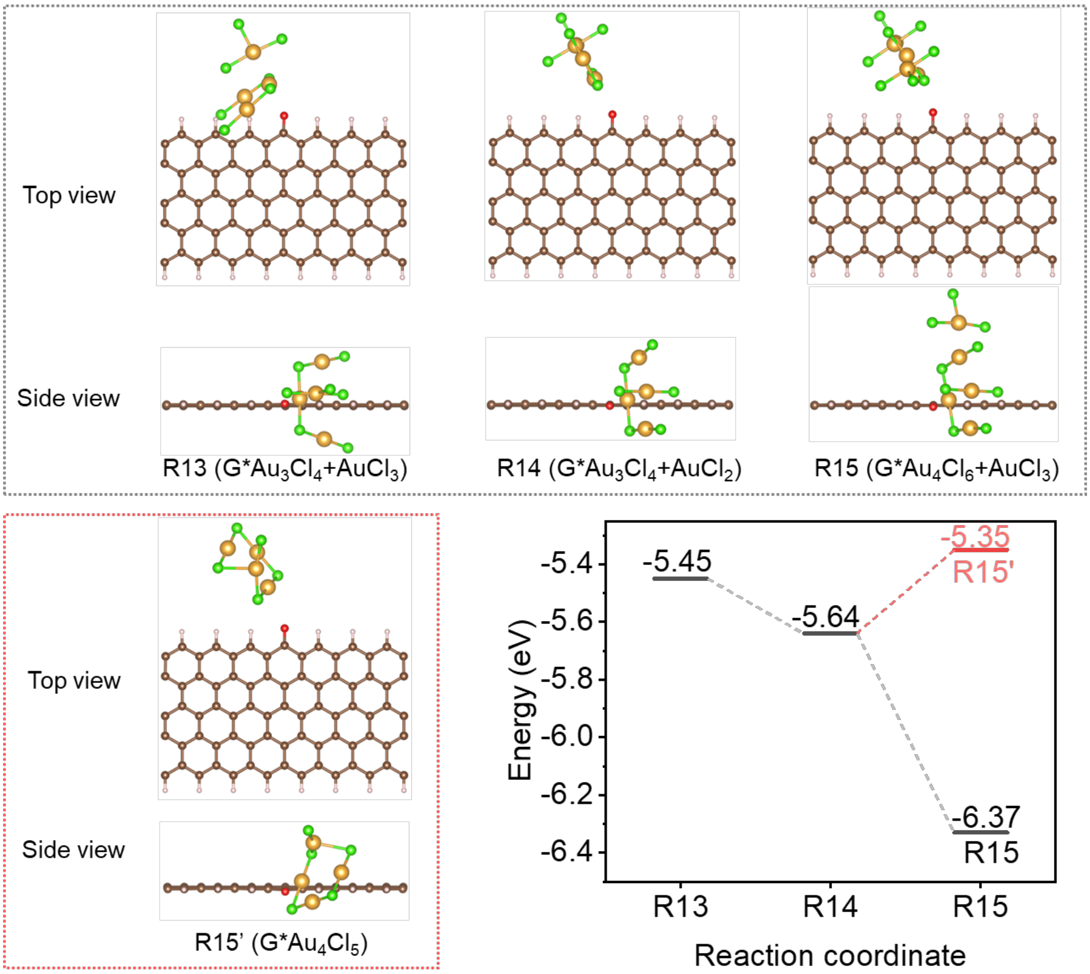

Supplementary Fig. 39. Schematic diagram and calculated energy diagram for the Au(III) ($AuCl_3$) adsorption and reduction on PyC700 from reaction coordinate 13 to 15 (R13 to R15) (R15' represents another possible coordination configuration after R14, however, it exhibits a higher reaction energy barrier compared to the optimal R15 we have inferred).

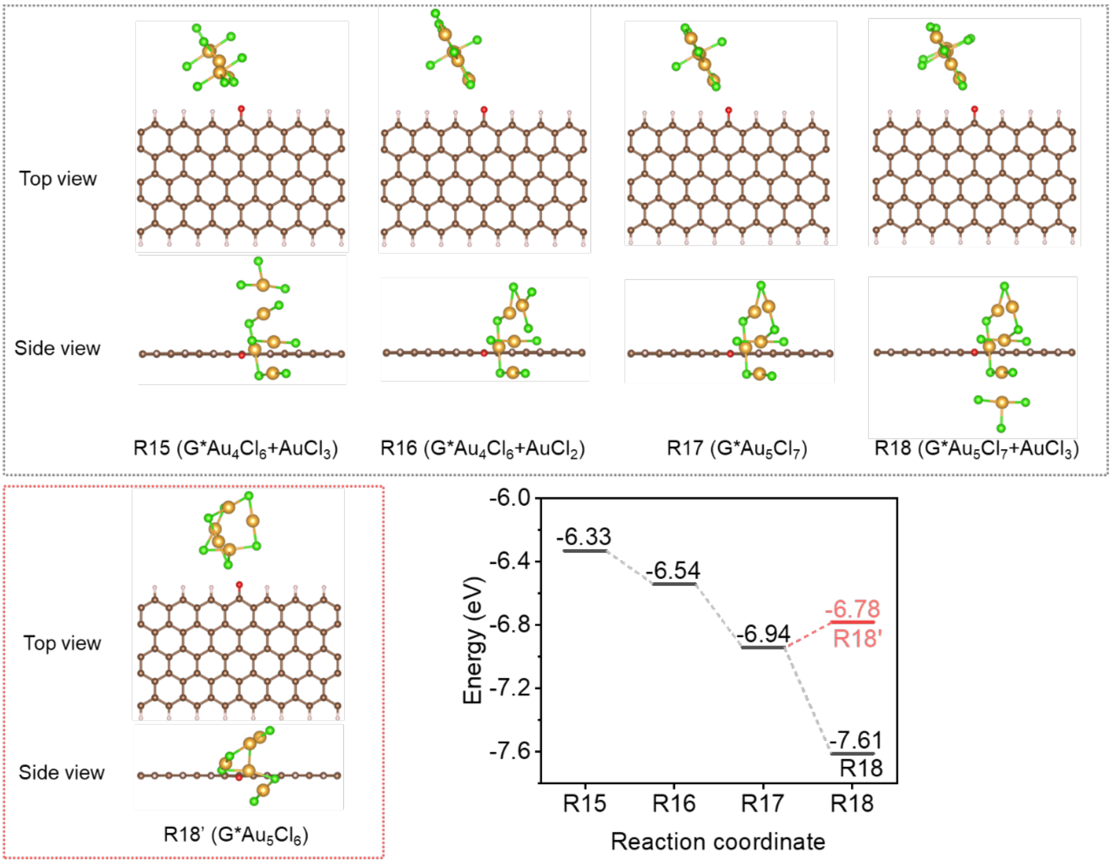

Supplementary Fig. 40. Schematic diagram and calculated energy diagram for the Au(III) ($AuCl_3$) adsorption and reduction on PyC700 from reaction coordinate 15 to 18 (R15 to R18) (R18' represents another possible coordination configuration after R17, however, it exhibits a higher reaction energy barrier compared to the optimal R18 we have inferred).

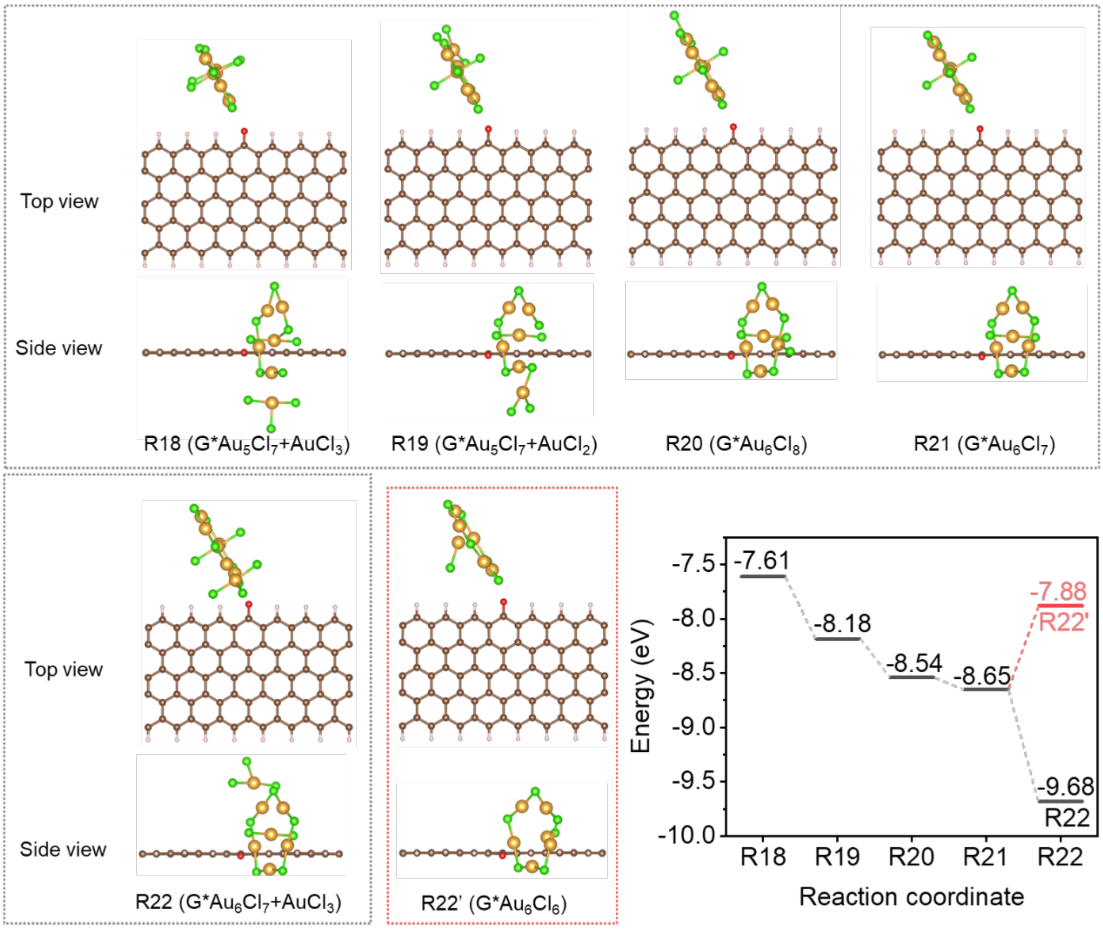

Supplementary Fig. 41. Schematic diagram and calculated energy diagram for the Au(III) ($AuCl_3$) adsorption and reduction on PyC700 from reaction coordinate 18 to 22 (R18 to R22) (R22' represents another possible coordination configuration after R21, however, it exhibits a higher reaction energy barrier compared to the optimal R22 we have inferred).

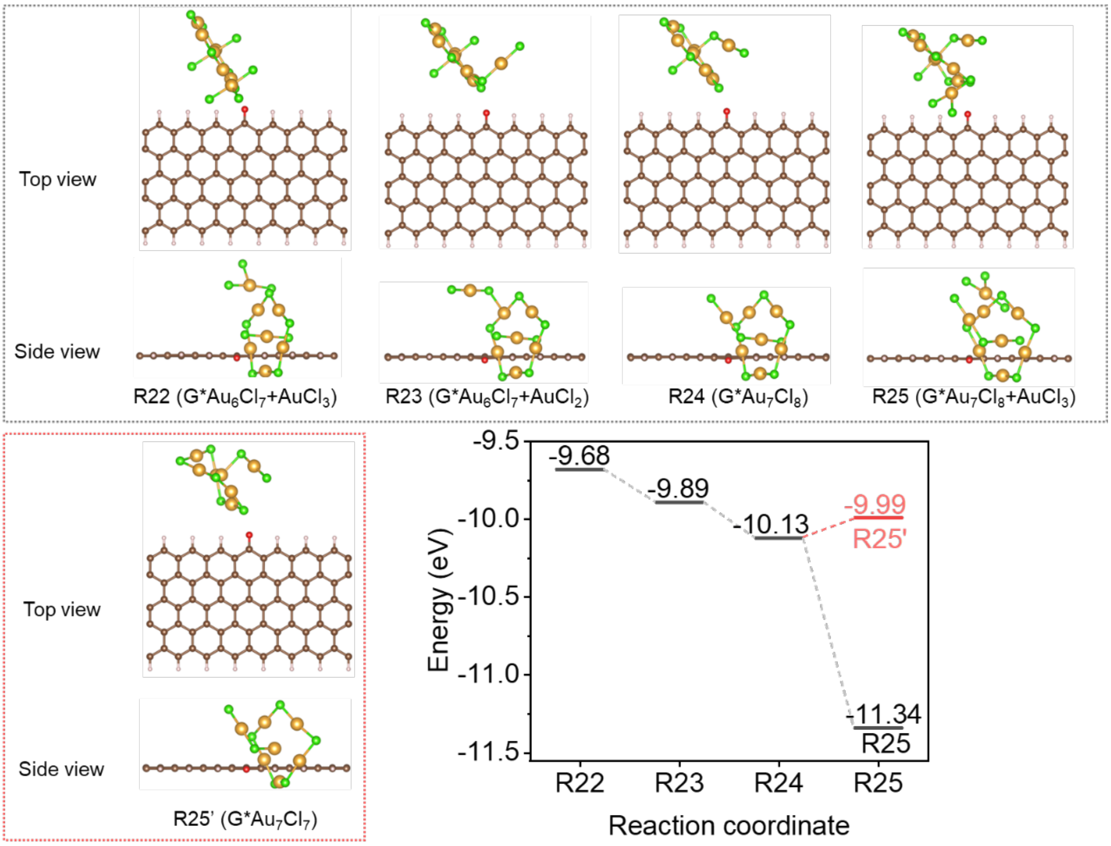

Supplementary Fig. 42. Schematic diagram and calculated energy diagram for the Au(III) ($AuCl_3$) adsorption and reduction on PyC700 from reaction coordinate 22 to 25 (R22 to R25) (R25' represents another possible coordination configuration after R24, however, it exhibits a higher reaction energy barrier compared to the optimal R25 we have inferred).

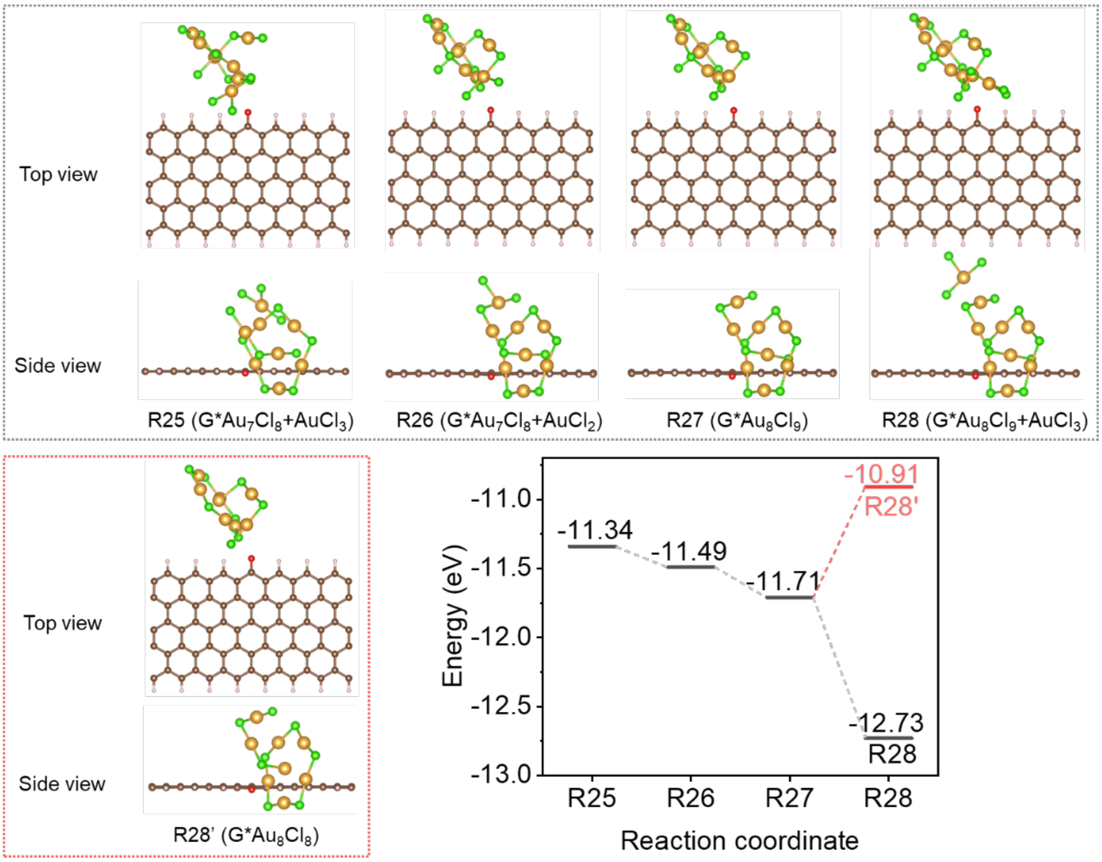

Supplementary Fig. 43. Schematic diagram and calculated energy diagram for the Au(III) ($AuCl_3$) adsorption and reduction on PyC700 from reaction coordinate 25 to 28 (R25 to R28) (R28' represents another possible coordination configuration after R27, however, it exhibits a higher reaction energy barrier compared to the optimal R28 we have inferred).

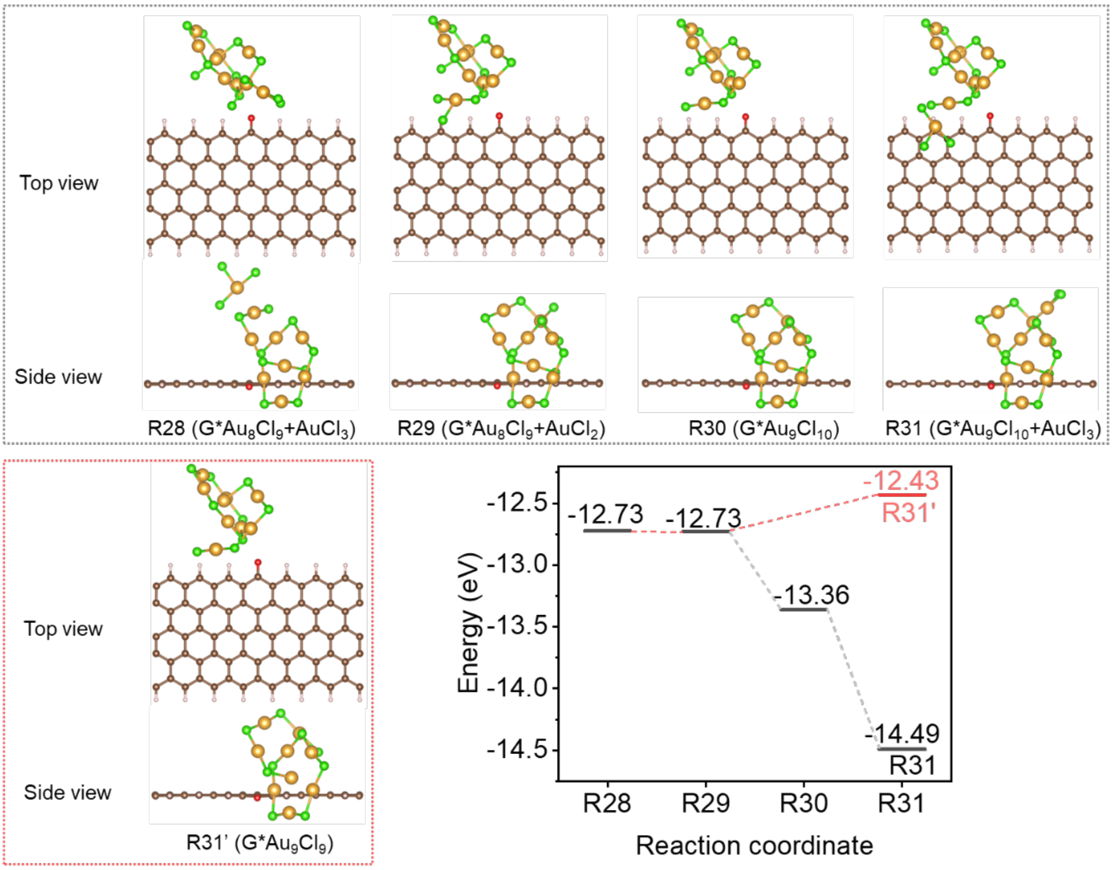

Supplementary Fig. 44. Schematic diagram and calculated energy diagram for the Au(III) ($AuCl_3$) adsorption and reduction on PyC700 from reaction coordinate 28 to 31 (R28 to R31) (R31' represents another possible coordination configuration after R30, however, it exhibits a higher reaction energy barrier compared to the optimal R31 we have inferred).

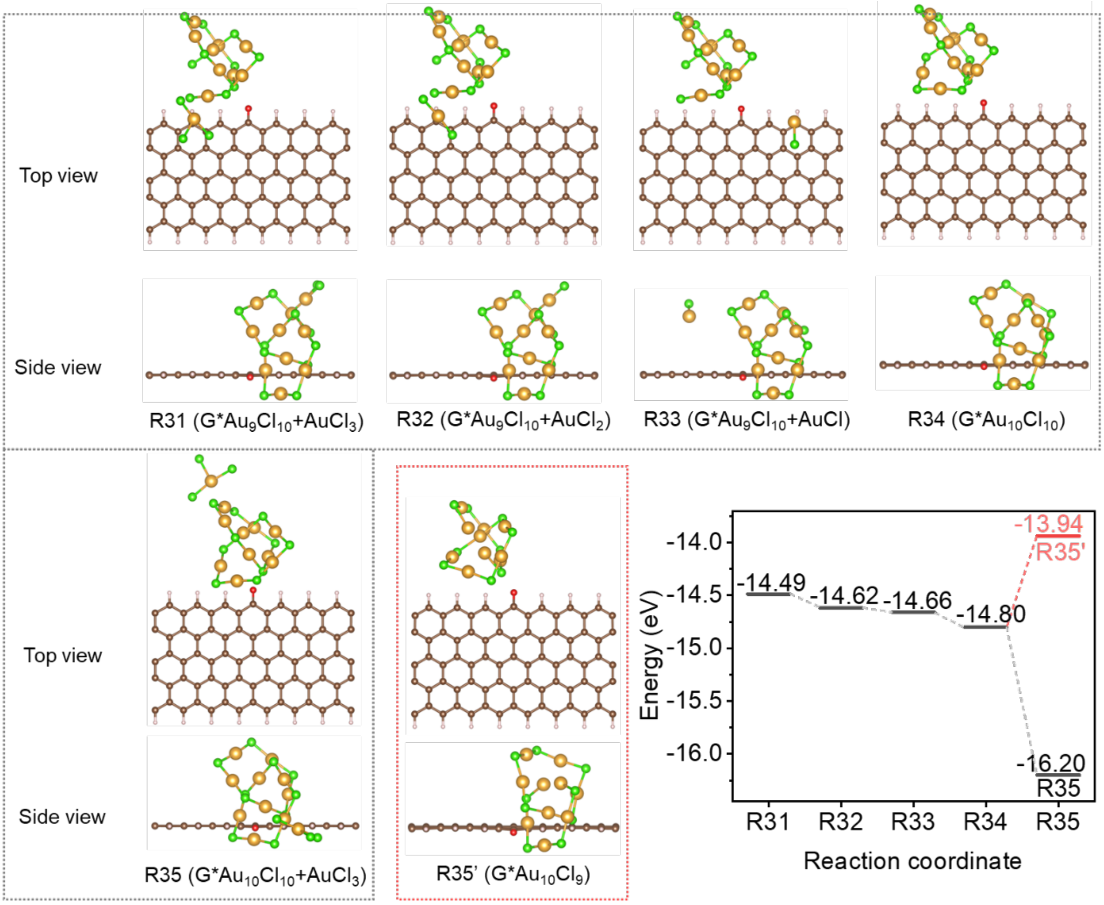

Supplementary Fig. 45. Schematic diagram and calculated energy diagram for the Au(III) ($AuCl_3$) adsorption and reduction on PyC700 from reaction coordinate 31 to 35 (R31 to R35) (R35' represents another possible coordination configuration after R34, however, it exhibits a higher reaction energy barrier compared to the optimal R35 we have inferred).

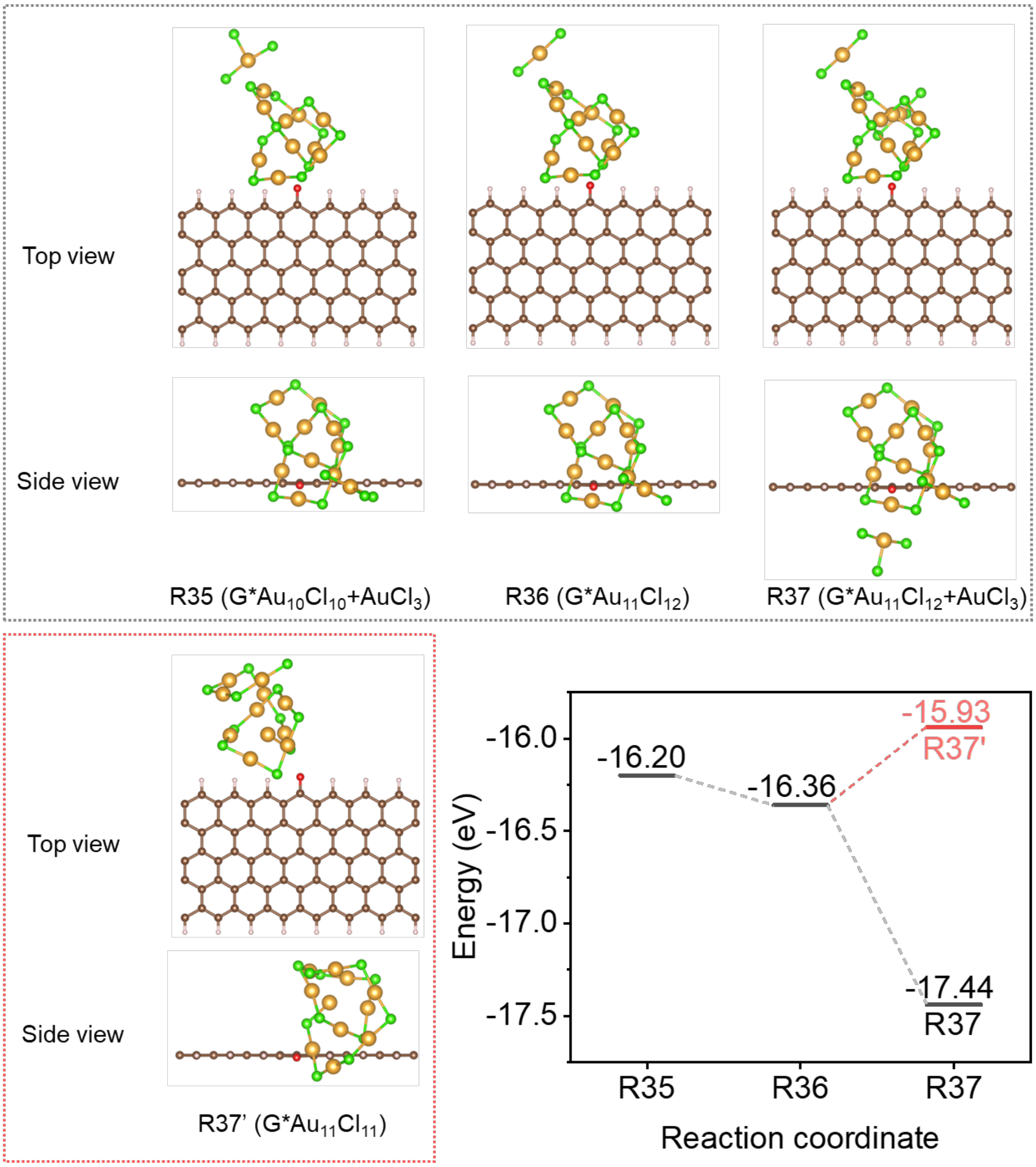

Supplementary Fig. 46. Schematic diagram and calculated energy diagram for the Au(III) ($AuCl_3$) adsorption and reduction on PyC700 from reaction coordinate 35 to 37 (R35 to R37) (R37' represents another possible coordination configuration after R36, however, it exhibits a higher reaction energy barrier compared to the optimal R37 we have inferred).

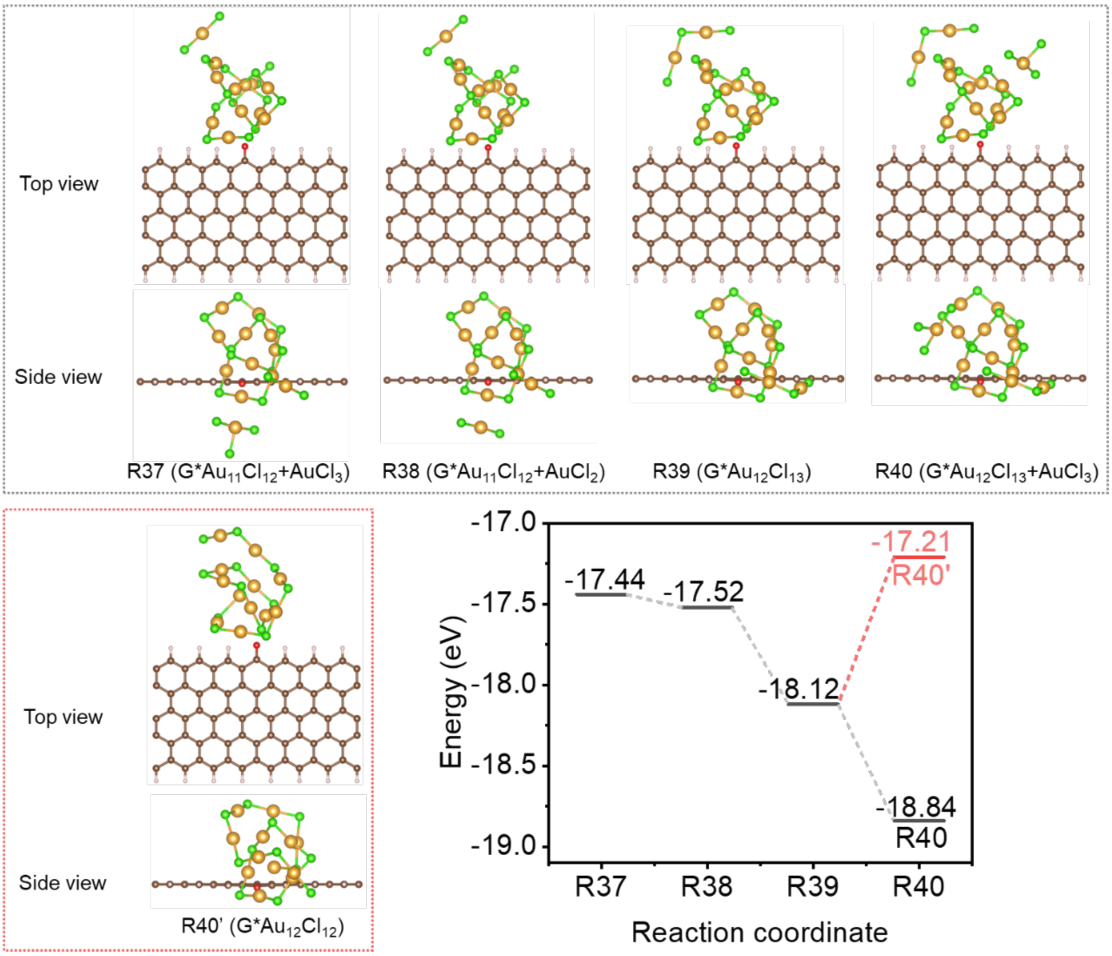

Supplementary Fig. 47. Schematic diagram and calculated energy diagram for the Au(III) ($AuCl_3$) adsorption and reduction on PyC700 from reaction coordinate 37 to 40 (R37 to R40) (R40' represents another possible coordination configuration after R39, however, it exhibits a higher reaction energy barrier compared to the optimal R40 we have inferred).

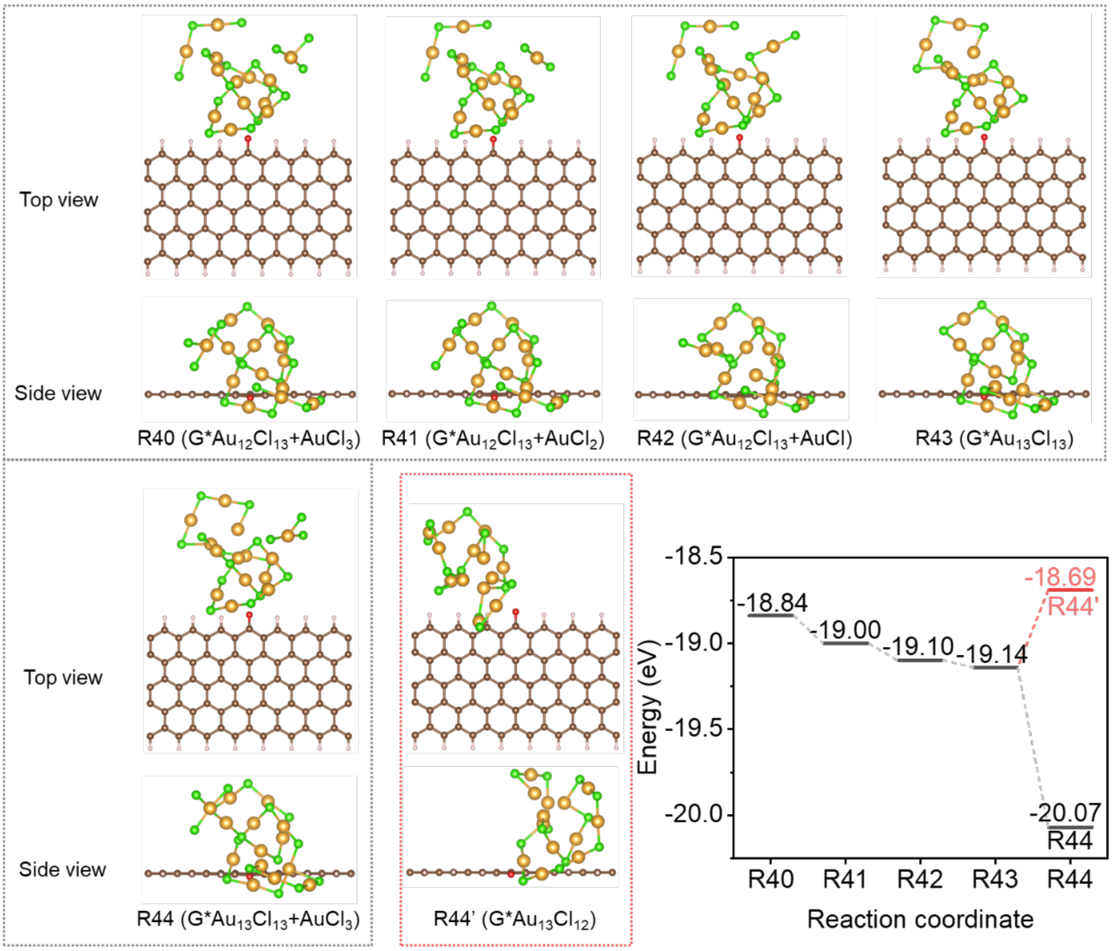

Supplementary Fig. 48. Schematic diagram and calculated energy diagram for the Au(III) ($AuCl_3$) adsorption and reduction on PyC700 from reaction coordinate 40 to 44 (R40 to R44) (R44' represents another possible coordination configuration after R43, however, it exhibits a higher reaction energy barrier compared to the optimal R44 we have inferred).

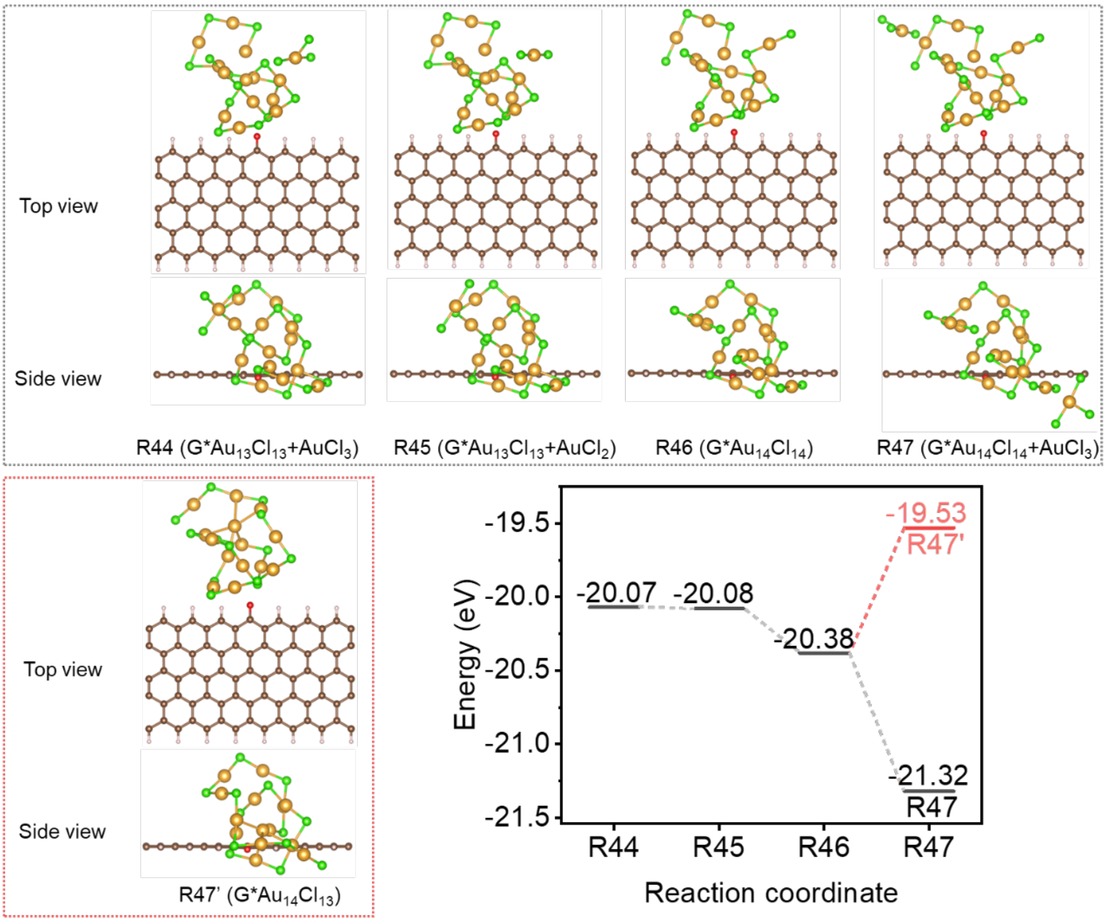

Supplementary Fig. 49. Schematic diagram and calculated energy diagram for the Au(III) ($AuCl_3$) adsorption and reduction on PyC700 from reaction coordinate 44 to 47 (R44 to R47) (R47' represents another possible coordination configuration after R46, however, it exhibits a higher reaction energy barrier compared to the optimal R47 we have inferred).

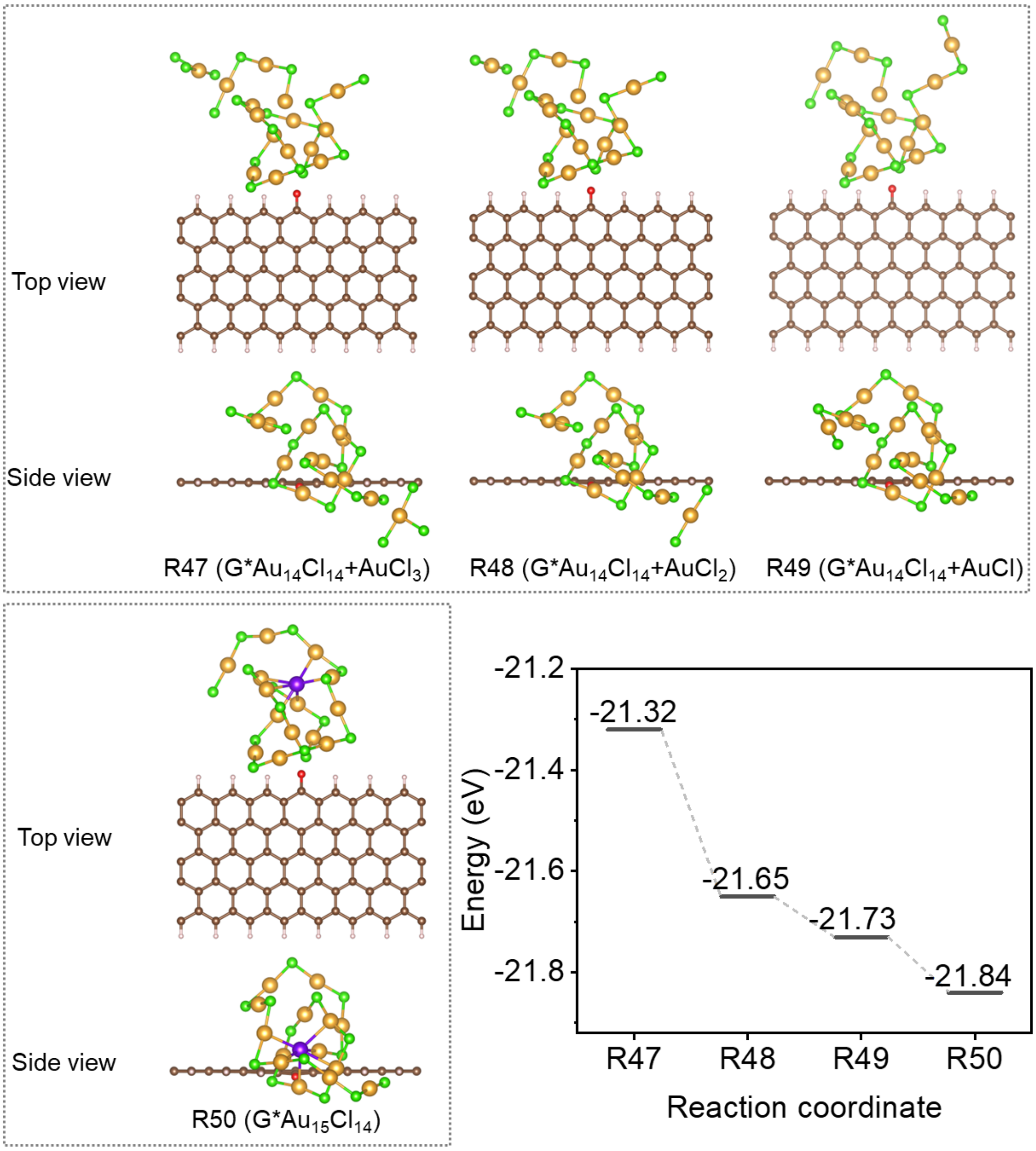

Supplementary Fig. 50. Schematic diagram and calculated energy diagram for the Au(III) ($AuCl_3$) adsorption and reduction on PyC700 from reaction coordinate 47 to 50 (R47 to R50). The center Au atom (marked with purple) only coordinate with six other Au atoms, indicating the complete reduction to zerovalent state Au^0.

a

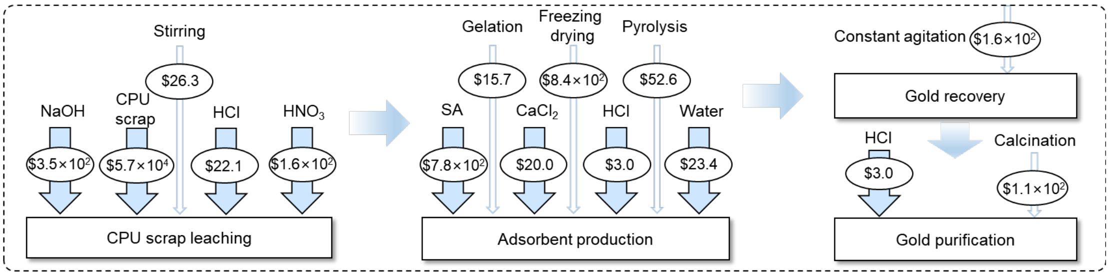

b

Equipment	Price (US $)	Power (kW)	Annual running time (h)
Water bath	27.43	0.3	730
Peristaltic pump	274.35	0.02	2190
Magnetic stirrer	20.58	0.2	2190
Freeze dryer	685.87	1.6	8760
Tube furnace	1646.09	1.2	2190
Thermostatic oscillator	548.70	0.38 ~ 0.60	4380
Glass wares	41.15	-	-

c

Chemicals and materials	Price (US $ ton^{-1})	Usage (ton)
NaOH	1194.70	0.291
Sodium alginate	10973.93	0.071
$CaCl_2$	68.58	0.292
HCl	40.47	0.693
HNO_3	205.76	0.755
Water	0.8	29.2
CPU scrap	13717.42	4.132

Supplementary Fig. 51. (a) Material flow analysis of the specific example of gold recovery process from CPU scrap using PyC700. The specific information of (b) the equipment and (c) the chemicals/materials involved in TEA for gold recovery based on PyC700.

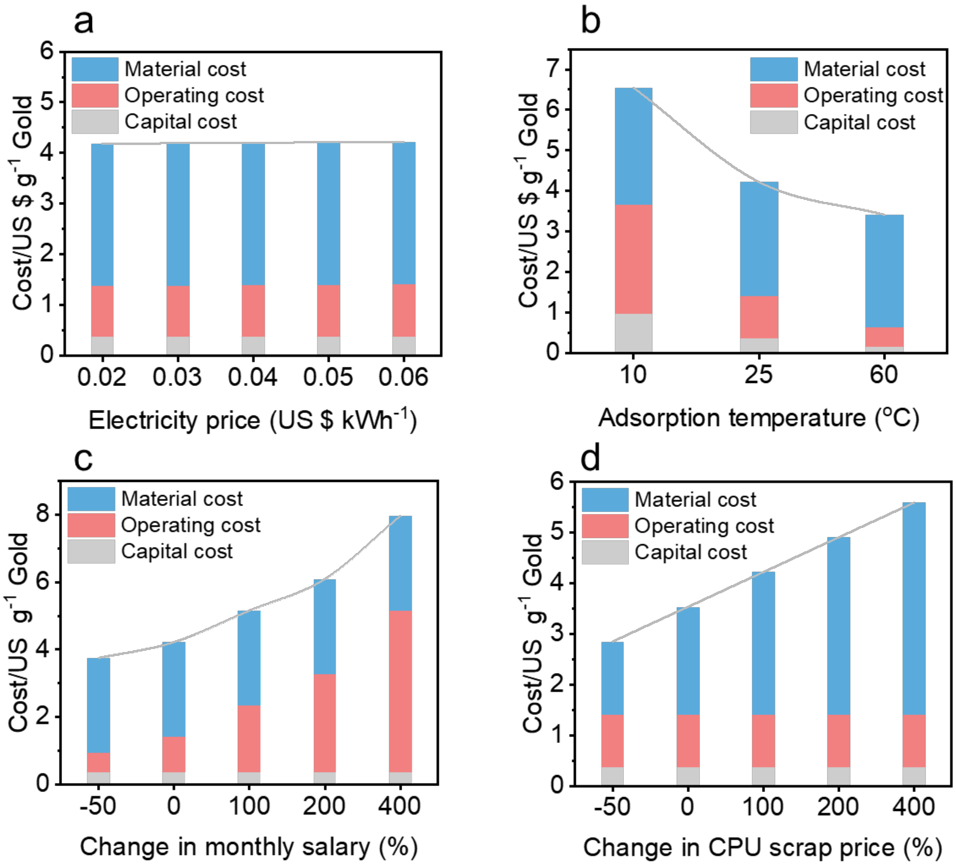

Supplementary Fig. 52. The cost distribution of gold recovery process by PyC700 under varying conditions including (a) Adsorption temperature, (b) Electricity price, (c) Monthly salary, and (d) CPU scrap price. Except for the specific variables in each

figure, other parameters used were set unchanged, with the adsorption temperature of 60ºC, electricity price of 0.06 \$ kWh^{-1}, change in monthly wage of 0%, change in in CPU waste price of 0%.

Supplementary Tables

Supplementary Table 1. The content of chemical bonds in pyrocarbon derived from XPS data and Boehm titration.

Sample	PyC500	PyC600	PyC700	PyC800
C=C, %[a]	40.1	46.9	54.2	58.4
C-C, %[a]	22.1	23.1	21.3	20.2
C-O, %[a]	32.6	23.2	16.3	14.6
O-C=O, %[a]	5.2	6.8	8.2	6.8
sp^2, %[a]	40.1	46.9	54.2	58.4
sp^3, %[a]	54.7	46.3	37.6	34.8
sp^2/sp^3 ratio[a]	0.73	1.02	1.42	1.66
-OH[b]	33.7	25.1	47.5	31.9
-COOH[b]	44.2	50.8	16.2	31.2
-C=O[b]	22.1	24.1	36.3	36.9
O/C atom ratio[b]	0.32	0.28	0.18	0.15
Phenolic -OH, mmol[c]	3.12	2.23	1.75	1.02

[a] The content of chemical bonds was calculated according to the corresponding quantity in high-resolution C 1*s* XPS spectra.

[b] The content of chemical bonds was calculated according to the corresponding quantity in high-resolution O 1*s* XPS spectra.

[c] The content of phenolic hydroxyl groups was determined using Boehm titration method.

Supplementary Table 2. Isotherm fitting of Langmuir model for Au(III) recovery processes by pyrocarbon.

Adsorbent	Temperature (°C)	Langmuir model		
		q_m (mg g^{-1})	k_L (L mg^{-1})	R^2
PyC500	25	1019.4	23.80	0.9940
PyC600	25	1604.1	15.36	0.9723
PyC700	10	1095.0	5.20	0.9411
	25	2829.7	5.72	0.9184
	60	6368.4	8.73	0.9563
PyC800	25	1947.7	23.53	0.9453

Supplementary Table 3. Calculation of mass transfer coefficient k_f for pyrocarbons.

Adsorbent	Effective area a (× 10^5 m^2 m^{-3})	Mass transfer coefficient k_f (× 10^{-8} m s^{-1})
PyC500	1.55	1.20
PyC600	1.64	1.21
PyC700	1.84	1.73
PyC800	2.55	0.89

Supplementary Table 4. Comparison of Au(III) recovery performance of previously reported adsorbents.

Gold adsorbent	T (°C)	pH	Q_m (mg g^{-1})	K_d (mL g^{-1})	Recovery mechanism	Reference
NH_2-UiO-66-BA	-	2.5	1040	-	S&R[a]	18
COF-HNU25	25	7	1725	6.9×10^6	S&R	19
JNM-100-AO	25	2	954	2.9×10^6	S [b]	20
DTDD-MOF	25	2	1119	-	S&R	21
PCN-225	25	3	2613	4.8×10^7	S&R	22
GCC51	25	2	882	1.4×10^4	S&R	23
UiO-66-TA	25	2	374.8	2.4×10^5	S&R	24
CNT-MoS_2(2H)	-	4.8	2495	5.6×10^5	S&R	25
porphyrin polymer	-	2	1540	-	S&R	26
UiO-66-TU	25	4	326	-	S&R	27
UiO-66-ATU	25	3	227.7	-	S&R	28
UiO-66-BTU	25	2.5	680	1.0×10^4	S&R	29
Fe-BTC/PpDDA	28	2	934	1.3×10^4	S&R	30
RS-SR-NH-SiO_2-Fe_3O_4	25	6	222	-	S	31
IM-TUCS	30	2	933	1.0×10^4	S	32
SH-MCM-41	20	2.5	195	-	S	33
UiO-66-NH_2	25	2.5	650	-	S&R	34
CuS NPs	25	1	574.7	3.1×10^6	S&R	35
MoS_2 NFs	25	1	1133	-	S&R	36
IECS-GLA	25	3	808.9	2.4×10^4	S	37
PGMA-NH_2	25	4	1623	-	S&R	38
Aginate beads	30	1	387.7	-	S&R	39
PEI-modified sorbent	25	1	285	-	S&R	40
Thiol-ene hydrogel	25	0.5	118.8	-	S	41
Diatom biochar	25	2.9	443	4.7×10^3	S&R	42
Barley straw carbon	30	-	289.6	-	S&R	43
Activated carbon fiber	-	-	49.3	-	S&R	44
Activated carbon	-	3	32.1	-	S&R	45
Commercia activated carbon	-	3	33	-	S&R	45

Gold adsorbent	T (°C)	pH	Q_m (mg g^{-1})	K_d (mL g^{-1})	Recovery mechanism	Reference
rGO	25	4	1850	-	S&R	6
GO	25	6	108	-	S	46
PT-GO	30	3	1325	-	S	47
POPAM-grafted MWCNT	25	3	97	-	S	48
MWCNT	-	-	93.5	-	S&R	49
PyC700	10	2.75	1095.0	-	S&R	This work
	25	2.75	2829.7	3.1×10^{8}	S&R	This work
	60	2.75	6368.4	-	S&R	This work

[a] The term “S&R” suggests that a sorption-coupled reduction mechanism governs the entire gold recovery process rather than simple sorption.
[b] The term “S” indicates that the gold recovery process relies solely on sorption processes without any valence change for enriched gold ions.

Supplementary Table 5. Thermodynamic parameters for Au(III) recovery by PyC700 at different temperatures.

T (°C)	ln K	ΔG^{o} (kJ mol^{-1})
10	17.86	-42.03
25	17.95	-44.50
60	18.37	-50.89
ΔH^{o} (kJ mol^{-1})	8.38	
ΔS^{o} (J (mol K)$^{-1}$)	177.75	

Supplementary Table 6. Isotherm fitting of Langmuir model for different gold ions adsorption by PyC700.

Gold ions species	Temperature (°C)	Langmuir model		
		q_m (mg g^{-1})	k_L (L mg^{-1})	R^2
$AuCl_4^-$	25	2829.7	5.72	0.9184
$AuBr_4^-$	25	934.9	0.22	0.9849
$Au(S_2O_3)_2^{3-}$	25	252.6	0.02	0.9426
$Au(CN)_2^-$	25	121.3	0.02	0.9326

Supplementary Table 7. Concentrations of typical cations in four types of leaching solutions of AMD and Intel CPUs.

Cations	AMD-Aqua	AMD-NBS	Intel-Aqua	Intel-NBS
Au(III)	1330	650.3	12.65	2.633
Na(I)	7.332	5.265	62.57	116.1
K(I)	1.136	1.577	1.949	9.591
Ca(II)	17.917	6.584	21.66	15.63
Mg(II)	2.616	1.576	2.852	3.333
Fe(III)	10108	578.6	102.6	39.67
Ni(II)	7035	157.6	2211	325.6
Cu(II)	29.55	22.5	33884	19839
Al(III)	35.24	28.79	1.919	1.157
Zn(II)	21.26	25.65	0.776	1.214
Mn(II)	1.072	1.781	1.525	2.129

Supplementary Table 8. The content of chemical bonds in PyC700 adsorption in Au(III) solution for varying times derived from XPS data.

Chemical composition	0.1 min	1 min	5 min	30 min
C=C, %[a]	41.3	30	28.2	22.9
C-C, %[a]	34.1	37.3	40.4	44.6
C-O, %[a]	15.6	25.7	22.3	20.3
O-C=O, %[a]	9	7	9.1	12.2
sp^2, %[a]	41.3	30	28.2	22.9
sp^3, %[a]	49.7	63	62.7	64.9
sp^2/sp^3 ratio[a]	0.83	0.48	0.45	0.35
-OH[b]	41.2	34.2	30.1	33.1
-COOH[b]	21.6	27.3	37.9	36.5
-C=O[b]	37.2	38.5	32	30.4
O/C atom ratio[b]	0.18	0.22	0.26	0.33

[a] The content of chemical bonds was calculated according to the corresponding quantity in high-resolution C 1*s* XPS spectra.

[b] The content of chemical bonds was calculated according to the corresponding quantity in high-resolution O 1*s* XPS spectra.

Supplementary Table 9. EXAFS curve fitting results of Au for Au-loaded PyC700 at sequential reaction time (0.1, 1, 5, and 30 min).

Sample	Shell	[a]CN	[b]R (Å)	[c]σ^2 (Å^2)	[d]ΔE_0 (eV)	[e]R factor
PyC700-Au (0.1 min)	Au-Au	6.5	2.86	0.0075	5.274	0.0213
	Au-Cl	0.47	2.27	0.001		
PyC700-Au (1 min)	Au-Au	6.79	2.86	0.0076	5.16	0.0113
	Au-Cl	0.46	2.25	0.0024		
PyC700-Au (5 min)	Au-Au	7.31	2.86	0.0081	4.926	0.0058
	Au-Cl	0.41	2.24	0.0029		
PyC700-Au (30 min)	Au-Au	7.57	2.86	0.0066	4.591	0.0156
	Au-Cl	0.19	2.25	0.001		

[a] CN is the coordination number.

[b] R is the atom distance (Å).

[c] σ^2 is the Debye-Waller factor (Å^2).

[d] ΔE_0 is the inner potential correction (eV).

[e] R factor indicates the goodness of the fit.

The amplitude reduction factor $S0^2$ was fixed to 0.900. Fitting range: $2.0 \leq k$ (Å) ≤ 12.5 and $1.5 \leq R$ (Å) ≤ 3.5.

Supplementary Table 10. The specific costs of the entire gold recovery process from CPU waste using PyC700.

Total costs	Specific items	Costs (US $ g^{-1} gold)	Percentage (%)
Capital costs (8.83%)	Equipment purchase cost	0.1570	3.72
	Other capital costs	0.2154	5.11
Operating costs (24.58%)	Electricity	0.0552	1.31
	Maintenance	0.0031	0.07
	Labor	0.6465	15.33
	Depreciation	0.0372	0.88
	Other operating cost	0.2947	6.99
Material costs (66.59)	NaOH	0.0169	0.40
	Sodium alginate	0.0375	0.89
	CPU waste	2.7435	65.04
	Other chemicals	0.0110	0.26

Supplementary Table 11. The variables of technological parameters in sensitivity analysis with assumed values from favorable to unfavorable.

Parameters	Values				
Electricity price (US$ kWh^{-1})	0.2	0.3	0.4	0.5	0.6
Adsorption temperature (ºC)	10		25		60
Monthly salary (US$ $month^{-1}$)	556.5	1113.1	2226.2	3339.2	5565.4
CPU scrap price (US$ ton^{-1})	6858.7	10288.1	13717.4	17146.8	20576.1

Supplementary References

1. Fu, K.; Luo, J., Rebuttal to Correspondence on "Superselective Hg (II) Removal from Water Using a Thiol-Laced MOF-Based Sponge Monolith: Performance and Mechanism". *Environ. Sci. Technol.* **2023,** *57* (36), 13543-13545.
2. Hu, W.; Yang, L.; Shao, P.; Shi, H.; Chang, Z.; Fang, D.; Wei, Y.; Feng, Y.; Huang, Y.; Yu, K.; Luo, X., Proton Self-Enhanced Hydroxyl-Enriched Cerium Oxide for Effective Arsenic Extraction from Strongly Acidic Wastewater. *Environ. Sci. Technol.* **2022,** *56* (14), 10412-10422.
3. Xiang, C.; Ji, Q.; Zhang, G.; Wang, H.; Qu, J., In Situ Creation of Oxygen Vacancies in Porous Bimetallic La/Zr Sorbent for Aqueous Phosphate: Hierarchical Pores Control Mass Transport and Vacancy Sites Determine Interaction. *Environ. Sci. Technol.* **2020,** *54* (1), 437-445.
4. Zhu, C.; Fang, Q.; Liu, R.; Dong, W.; Song, S.; Shen, Y., Insights into the Crucial Role of Electron and Spin Structures in Heteroatom-Doped Covalent Triazine Frameworks for Removing Organic Micropollutants. *Environ. Sci. Technol.* **2022,** *56* (10), 6699-6709.
5. Wang, D.; Ma, J.; Zhang, J.; Strathmann, T. J., Carbocatalysts for Enhancing Permanganate Oxidation of Sulfisoxazole. *Environ. Sci. Technol.* **2023**.
6. Li, F.; Zhu, J.; Sun, P.; Zhang, M.; Li, Z.; Xu, D.; Gong, X.; Zou, X.; Geim, A.; Su, Y., Highly efficient and selective extraction of gold by reduced graphene oxide. *Nat. Commun.* **2022,** *13* (1), 4472.
7. Danaci, D.; Webley, P. A.; Petit, C., Guidelines for techno-economic analysis of adsorption processes. *Frontiers in Chemical Engineering* **2021,** *2*, 602430.
8. Zhao, B.-H.; Chen, F.; Wang, M.; Cheng, C.; Wu, Y.; Liu, C.; Yu, Y.; Zhang, B., Economically viable electrocatalytic ethylene production with high yield and selectivity. *Nat. Sustain.* **2023**, 1-11.
9. Wang, J.; Jia, K.; Ma, J.; Liang, Z.; Zhuang, Z.; Zhao, Y.; Li, B.; Zhou, G.; Cheng, H.-M., Sustainable upcycling of spent LiCoO2 to an ultra-stable battery cathode at high voltage. *Nat. Sustain.* **2023**, 1-9.
10. Arshadi, M.; Yaghmaei, S.; Mousavi, S., Content evaluation of different waste PCBs to enhance basic metals recycling. *Resour. Conserv. Recycl.* **2018,** *139*, 298-306.
11. Huang, T.; Zhu, J.; Huang, X.; Ruan, J.; Xu, Z., Assessment of precious metals positioning in waste printed circuit boards and the economic benefits of recycling. *Waste Manag.* **2022,** *139*, 105-115.
12. Yunus, Z. M.; Al-Gheethi, A.; Othman, N.; Hamdan, R.; Ruslan, N. N., Removal of heavy metals from mining effluents in tile and electroplating industries

using honeydew peel activated carbon: A microstructure and techno-economic analysis. *J. Clean. Prod.* **2020,** *251*, 119738.
13. Wright, M. M.; Daugaard, D. E.; Satrio, J. A.; Brown, R. C., Techno-economic analysis of biomass fast pyrolysis to transportation fuels. *Fuel* **2010,** *89*, S2-S10.
14. Elhafez, S. A.; Hamad, H.; Zaatout, A.; Malash, G., Management of agricultural waste for removal of heavy metals from aqueous solution: adsorption behaviors, adsorption mechanisms, environmental protection, and techno-economic analysis. *Environ. Sci. Pollut. R.* **2017,** *24*, 1397-1415.
15. Wang, M.; Ren, T.; Yin, M.; Lu, K.; Xu, H.; Huang, X.; Zhang, X., Enhanced Anaerobic Wastewater Treatment by a Binary Electroactive Material: Pseudocapacitance/Conductance-Mediated Microbial Interspecies Electron Transfer. *Environ. Sci. Technol.* **2023,** *57* (32), 12072-12082.
16. Thanh, N. T. K.; Maclean, N.; Mahiddine, S., Mechanisms of nucleation and growth of nanoparticles in solution. *Chem. Rev.* **2014,** *114* (15), 7610-7630.
17. Yang, T.-H.; Zhou, S.; Gilroy, K. D.; Figueroa-Cosme, L.; Lee, Y.-H.; Wu, J.-M.; Xia, Y., Autocatalytic surface reduction and its role in controlling seed-mediated growth of colloidal metal nanocrystals. *Proc. Natl. Acad. Sci. U.S.A.* **2017,** *114* (52), 13619-13624.
18. Cao, J.; Xu, Z.; Chen, Y.; Li, S.; Jiang, Y.; Bai, L.; Yu, H.; Li, H.; Bian, Z., Tailoring the Asymmetric Structure of NH2-UiO-66 Metal-Organic Frameworks for Light-promoted Selective and Efficient Gold Extraction and Separation. *Angew. Chem.* **2023,** *135* (18), e202302202.
19. Qiu, J.; Xu, C.; Xu, X.; Zhao, Y.; Zhao, Y.; Zhao, Y.; Wang, J., Porous Covalent Organic Framework Based Hydrogen-Bond Nanotrap for the Precise Recognition and Separation of Gold. *Angew. Chem.* **2023,** *135* (17), e202300459.
20. Luo, J.; Luo, X.; Xie, M.; Li, H.-Z.; Duan, H.; Zhou, H.-G.; Wei, R.-J.; Ning, G.-H.; Li, D., Selective and rapid extraction of trace amount of gold from complex liquids with silver (I)-organic frameworks. *Nat. Commun.* **2022,** *13* (1), 7771.
21. Huang, Z.; Zhao, M.; Wang, C.; Wang, S.; Dai, L.; Zhang, L.; Xu, L., Selective removal mechanism of the novel Zr-based metal organic framework adsorbents for gold ions from aqueous solutions. *Chem. Eng. J.* **2020,** *384*, 123343.
22. Shu, Y.; Chen, Y.; Han, Q.; Liu, X.; Liu, B.; Wang, Z., Selective and Light-Enhanced Au (III) Recovery by a Porphyrin-Based Metal–Organic Framework: Performance and Underlying Mechanisms. *ACS ES&T Engineering* **2023,** *3* (7), 1042-1052.
23. Bui, T. H.; Lee, W.; Jeon, S.-B.; Kim, K.-W.; Lee, Y., Enhanced Gold (III)

adsorption using glutaraldehyde-crosslinked chitosan beads: Effect of crosslinking degree on adsorption selectivity, capacity, and mechanism. *Sep. Purif. Technol.* **2020,** *248*, 116989.
24. Wang, C.; Lin, G.; Zhao, J.; Wang, S.; Zhang, L.; Xi, Y.; Li, X.; Ying, Y., Highly selective recovery of Au (III) from wastewater by thioctic acid modified Zr-MOF: Experiment and DFT calculation. *Chem. Eng. J.* **2020,** *380*, 122511.
25. Liu, F.; You, S.; Wang, Z.; Liu, Y., Redox-active nanohybrid filter for selective recovery of gold from water. *ACS ES&T Engineering* **2021,** *1* (9), 1342-1350.
26. Hong, Y.; Thirion, D.; Subramanian, S.; Yoo, M.; Choi, H.; Kim, H. Y.; Stoddart, J. F.; Yavuz, C. T., Precious metal recovery from electronic waste by a porous porphyrin polymer. *Proc. Natl. Acad. Sci. U.S.A.* **2020,** *117* (28), 16174-16180.
27. Wu, C.; Zhu, X.; Wang, Z.; Yang, J.; Li, Y.; Gu, J., Specific recovery and in situ reduction of precious metals from waste to create MOF composites with immobilized nanoclusters. *Ind. Eng. Chem. Res.* **2017,** *56* (47), 13975-13982.
28. Zhao, M.; Huang, Z.; Wang, S.; Zhang, L.; Wang, C., Experimental and DFT study on the selective adsorption mechanism of Au (III) using amidinothiourea-functionalized UiO-66-NH2. *Microporous Mesoporous Mater.* **2020,** *294*, 109905.
29. Guo, J.; Fan, X.; Wang, J.; Yu, S.; Laipan, M.; Ren, X.; Zhang, C.; Zhang, L.; Li, Y., Highly efficient and selective recovery of Au (III) from aqueous solution by bisthiourea immobilized UiO-66-NH2: Performance and mechanisms. *Chem. Eng. J.* **2021,** *425*, 130588.
30. Sun, D. T.; Gasilova, N.; Yang, S.; Oveisi, E.; Queen, W. L., Rapid, selective extraction of trace amounts of gold from complex water mixtures with a metal–organic framework (MOF)/polymer composite. *J. Am. Chem. Soc.* **2018,** *140* (48), 16697-16703.
31. Abd Razak, N. F.; Shamsuddin, M.; Lee, S. L., Adsorption kinetics and thermodynamics studies of gold (III) ions using thioctic acid functionalized silica coated magnetite nanoparticles. *Chem. Eng. Res. Des.* **2018,** *130*, 18-28.
32. Guo, J.; Fan, X.; Li, Y.; Yu, S.; Zhang, Y.; Wang, L.; Ren, X., Mechanism of selective gold adsorption on ion-imprinted chitosan resin modified by thiourea. *J. Hazard. Mater.* **2021,** *415*, 125617.
33. Lam, K. F.; Fong, C. M.; Yeung, K. L.; Mckay, G., Selective adsorption of gold from complex mixtures using mesoporous adsorbents. *Chem. Eng. J.* **2008,** *145* (2), 185-195.
34. Chang, Z.; Li, F.; Qi, X.; Jiang, B.; Kou, J.; Sun, C., Selective and efficient adsorption of Au (III) in aqueous solution by Zr-based metal-organic

frameworks (MOFs): An unconventional way for gold recycling. *J. Hazard. Mater.* **2020,** *391*, 122175.
35. Yao, C.; Chen, S.; Wang, L.; Deng, H.; Tong, S., Low cost and rapid fabrication of copper sulfides nanoparticles for selective and efficient capture of noble metal ions. *Chem. Eng. J.* **2019,** *373*, 1168-1178.
36. Feng, B.; Yao, C.; Chen, S.; Luo, R.; Liu, S.; Tong, S., Highly efficient and selective recovery of Au (III) from a complex system by molybdenum disulfide nanoflakes. *Chem. Eng. J.* **2018,** *350*, 692-702.
37. Ahamed, M.; Mbianda, X.; Mulaba-Bafubiandi, A.; Marjanovic, L., Selective extraction of gold (III) from metal chloride mixtures using ethylenediamine N-(2-(1-imidazolyl) ethyl) chitosan ion-imprinted polymer. *Hydrometallurgy* **2013,** *140*, 1-13.
38. Wang, F.-C.; Zhao, J.-M.; Wang, W.-K.; Tong, Z.-Z., Adsorption of Au (III) by amino-modified monodispersed PGMA microspheres and deposition of gold nanoparticles. *Rare Metals* **2018,** *37*, 196-203.
39. Gao, X.; Zhang, Y.; Zhao, Y., Zinc oxide templating of porous alginate beads for the recovery of gold ions. *Carbohydr. Polym.* **2018,** *200*, 297-304.
40. Choudhary, B. C.; Paul, D.; Borse, A. U.; Garole, D. J., Surface functionalized biomass for adsorption and recovery of gold from electronic scrap and refinery wastewater. *Sep. Purif. Technol.* **2018,** *195*, 260-270.
41. Fırlak, M.; Yetimoğlu, E. K.; Kahraman, M. V., Adsorption of Au (III) ions from aqueous solutions by thiol-ene photoclick hydrogels and its application to electronic waste and geothermal water. *J. Water Process Eng.* **2014,** *3*, 105-116.
42. Wang, L.; Li, J.; Zhong, G.; Li, J.; Lu, X.; Wang, S.; Tang, Y., Diatom Biochar Recovered Au(III) Efficiently from Both Synthetic and Real Electroplating Wastewaters. *ACS ES&T Water* **2023,** *3* (5), 1395-1405.
43. Chand, R.; Watari, T.; Inoue, K.; Kawakita, H.; Luitel, H. N.; Parajuli, D.; Torikai, T.; Yada, M., Selective adsorption of precious metals from hydrochloric acid solutions using porous carbon prepared from barley straw and rice husk. *Miner. Eng.* **2009,** *22* (15), 1277-1282.
44. Simanova, S.; Shukarev, A.; Lysenko, A.; Grebennikov, S.; Astashkina, O., Adsorption of palladium, platinum, and gold chloride complexes by carbon fibers with various structures. *Fibre chemistry* **2008,** *40* (4).
45. Buah, W. K.; Williams, P. T., Activated carbons prepared from refuse derived fuel and their gold adsorption characteristics. *Environ. Technol.* **2010,** *31* (2), 125-137.
46. Liu, L.; Liu, S.; Zhang, Q.; Li, C.; Bao, C.; Liu, X.; Xiao, P., Adsorption of Au(III), Pd(II), and Pt(IV) from Aqueous Solution onto Graphene Oxide. *J. Chem. Eng. Data* **2012,** *58* (2), 209-216.

47. Wang, Z.; Li, X.; Liang, H.; Ning, J.; Zhou, Z.; Li, G., Equilibrium, kinetics and mechanism of Au^{3+}, Pd^{2+} and Ag^{+} ions adsorption from aqueous solutions by graphene oxide functionalized persimmon tannin. *Mater. Sci. Eng. C* **2017,** *79*, 227-236.
48. Behbahani, M.; Gorji, T.; Mahyari, M.; Salarian, M.; Bagheri, A.; Shaabani, A., Application of Polypropylene Amine Dendrimers (POPAM)-Grafted MWCNTs Hybrid Materials as a New Sorbent for Solid-Phase Extraction and Trace Determination of Gold(III) and Palladium(II) in Food and Environmental Samples. *Food Anal. Method.* **2013,** *7* (5), 957-966.
49. Pang, S.-K.; Yung, K.-C., Prerequisites for achieving gold adsorption by multiwalled carbon nanotubes in gold recovery. *Chem. Eng. Sci.* **2014,** *107*, 58-65.
