# Supplementary material for: Utilizing cost-effective pyrocarbon for highly efficient gold retrieval from e-waste leachate
Source: Nat Commun. 2024 Jul 20;15:6137. doi: 10.1038/s41467-024-50595-4 (PMC11271467; doi:10.1038/s41467-024-50595-4)
Supplement: Supplementary file 3 — Description of Additional Supplementary Files [file 41467_2024_50595_MOESM3_ESM.pdf]

Description of Additional Supplementary Files

File Name: Supplementary Data 1
Description: The optimized structure for Au0-loaded PyC700 from DFT calculations
